# Supplementary material for: Availability, prices and affordability of essential medicines in Zhejiang Province, China
Source: PLoS One. 2020 Nov 24;15(11):e0241761. doi: 10.1371/journal.pone.0241761 (PMC7685453; doi:10.1371/journal.pone.0241761)
Supplement: S1 File — (ZIP) [file pone.0241761.s001.zip › PLOS ONE Manuscript research data/Research data/Arts and Science Hospital.docx]

Availability of essential drugs in Zhejiang Province

Note: 1**All blanks**

2. **Package specification**Refers to the total number of packages in a single box, e.g200Press,100Granules (tablets), etc.If there is no recommended package specification, please select the maximum package size of the drug in your company while ensuring that the dosage form and specification remain unchanged.

3. **tablet**It means that the dosage form of the drug can be either tablet or capsule.

4. **Minimum unit price:**For the original research drug, the minimum unit price refers to the minimum unit price of the drug under the determined dosage form, specification, packaging specification, trade name and manufacturer; for generic drugs, the minimum unit price refers to the minimum unit price of the drug under the determined dosage form, specification and packaging specification.

**Usage questionnaire ()**Contact person: Wang Jialiang, director of Pharmacy Department

| Serial number | Common name  Specifications  Dosage form | category | Trade name | Manufacturer | Should I  drugs | Suggestion package  Installation specification | Our company  Installation specification | The package specification price | Minimum order  Bit price |
| --- | --- | --- | --- | --- | --- | --- | --- | --- | --- |
| 1 | Salbutamol sulfate  100ug / press  Inhaled aerosol | Original drug | ventolin | GlaxoSmithKline | Yes (√)  None () | 200Press (spray) | 200Press | 20.69 | 0.10345 |
|  |  | Anda |  |  | Yes ()  None (√) | 200Press (spray) |  |  |  |
| 2 | Metformin hydrochloride  500mg / capsule  Tablets / capsules | Original drug | Gehuazhi | Bristol Myers Squibb | Yes (√)  None () | 100Grains (tablets) | 20slice | 22.97 | 1.1485 |
|  |  | Anda |  |  | Yes ()  None (√) | 100Grains (tablets) |  |  |  |
| 3 | Bisoprolol fumarate  5mg / capsule  Tablets / capsules | Original drug | Kangke | Merck | Yes ()  None (√) | 60Grains (tablets) |  |  |  |
|  |  | Anda |  | Chengdu Yuandong biopharmaceutical Co., Ltd | Yes (√)  None () | 60Grains (tablets) | 2.5mg * 18 tablets | 23.33 | 2.59 |
| 4 | captopril  25mg / capsule  Tablets / capsules | Original drug | Caputon | Bristol Myers Squibb | Yes ()  None (√) | 60Grains (tablets) |  |  |  |
|  |  | Anda |  | Zhejiang deende Pharmaceutical Co., Ltd | Yes (√)  None () | 60Grains (tablets) | 100slice | 2.4 | 0.024 |
| 5 | Simvastatin  20mg / capsule  Tablets / capsules | Original drug | Shujiangzhi | Mershadong | Yes ()  None (√) | 30Grains (tablets) |  |  |  |
|  |  | Anda | Jingbi Shuxin | Zhejiang Jingxin Pharmaceutical Co., Ltd | Yes (√)  None () | 30Grains (tablets) | 14slice | 9.95 | 0.711 |
| 6 | Amitriptyline hydrochloride  25mg / capsule  Tablets / capsules | Original drug | Tryptizol | Mershadong | Yes ()  None (√) | 100Grains (tablets) |  |  |  |
|  |  | Anda |  | Hunan Dongting Pharmaceutical Co., Ltd | Yes (√)  None () | 100Grains (tablets) | 100slice | 16.84 | 0.1684 |
| 7 | ciprofloxacin  500mg / capsule  Tablets / capsules | Original drug | Sipple | Bayer | Yes ()  None (√) | 10Grains (tablets) |  |  |  |
|  |  | Anda |  |  | Yes ()  None (√) | 10Grains (tablets) |  |  |  |
| 8 | Compound sulfamethoxazole  8+40mg/ml  Suspension | Original drug | Bactrim | Roche | Yes ()  None (√) | 100ml |  |  |  |
|  |  | Anda |  |  | Yes ()  None (√) | 100ml |  |  |  |

Shaoxing district hospital: (Shaoxing district hospital)

| Serial number | Common name  Specifications  Dosage form | category | Trade name | Manufacturer | Should I  drugs | Suggestion package  Installation specification | Our company  Installation specification | The package specification price | Minimum order  Bit price |
| --- | --- | --- | --- | --- | --- | --- | --- | --- | --- |
| 9 | Amoxicillin  500mg / capsule  Tablets / capsules | Original drug | Amoxil | GlaxoSmithKline | Yes (√)  None () | 21Grains (tablets) | 20grain | 12.36 | 0.618 |
|  |  | Anda |  |  | Yes ()  None (√) | 21Grains (tablets) |  |  |  |
| 10 | Ceftriaxone sodium  1g / piece  Injections | Original drug | Rocephin | Roche | Yes (√)  None () | 1branch | 1branch | 51.34 | 51.34 |
|  |  | Anda |  | Taiwan Pansheng Pharmaceutical Co., Ltd | Yes (√)  None () | 1branch | 2G * 1 piece | 53.78 | 26.89 |
| 11 | omeprazole  20mg / capsule  Tablets / capsules | Original drug | Losec | AstraZeneca | Yes ()  None (√) | 30Grains (tablets) |  |  |  |
|  |  | Anda | Jinaokang | Zhejiang Jinhua Kangenbei | Yes (√)  None () | 30Grains (tablets) | 14grain | 54.84 | 3.917 |
| 12 | diazepam  5mg / capsule  Tablets / capsules | Original drug | Valium | Roche | Yes ()  None (√) | 100Grains (tablets) |  |  |  |
|  |  | Anda |  | Shandong Xinyi Pharmaceutical Co., Ltd | Yes (√)  None () | 100Grains (tablets) | 20slice | 4.74 | 0.237 |
| 13 | Oseltamivir  75mg / capsule  Tablets / capsules | Original drug | Tamiflu? | Roche | Yes ()  None (√) | 100Grains (tablets) | 10grain | 219.31 | 21.931 |
|  |  | Anda | Kewei | Yichang dongyangguang Changjiang Pharmaceutical Co., Ltd | Yes (√)  None () | 100Grains (tablets) | 10grain | 137.58 | 13.758 |
| 14 | Paracetamol  500mg / capsule  Tablets / capsules | Original drug | Billiton | GlaxoSmithKline | Yes (√)  None () | 10Grains (tablets) | 10slice | 4.56 | 0.456 |
|  |  | Anda |  |  | Yes ()  None (√) | 10Grains (tablets) |  |  |  |
| 15 | diclofenac sodium  25mg / capsule  Tablets / capsules | Original drug | Votalin | Novartis | Yes (√)  None () | 30Grains (tablets) | 30slice | 16.96 | 0.564 |
|  |  | Anda |  |  | Yes ()  None (√) | 30Grains (tablets) |  |  |  |
| 16 | Atenolol  50mg / capsule  Tablets / capsules | Original drug | Tinomin | AstraZeneca | Yes ()  None (√) | 60Grains (tablets) |  |  |  |
|  |  | Anda |  |  | Yes ()  None (√) | 60Grains (tablets) |  |  |  |

| Serial number | Common name  Specifications  Dosage form | category | Trade name | Manufacturer | Should I  drugs | Suggestion package  Installation specification | Our company  Installation specification | The package specification price | Minimum order  Bit price |
| --- | --- | --- | --- | --- | --- | --- | --- | --- | --- |
| 17 | Glimepiride  2mg / capsule  Tablets / capsules | Original drug | Amaryl | Sanofi Aventis | Yes (√)  None () | 15Grains (tablets) | 15slice | 64.31 | 4.287 |
|  |  | Anda |  |  | Yes ()  None (√) | 15Grains (tablets) |  |  |  |
| 18 | Clarithromycin  250mg / capsule  Tablets / capsules | Original drug | Krashen | Abbott | Yes ()  None (√) | 12Grains (tablets) |  |  |  |
|  |  | Anda | Nobond | Jiangsu Hengrui Pharmaceutical Co., Ltd | Yes (√)  None () | 12Grains (tablets) | 0.5g * 3 tablets | 12.6 | 2.1 |
| 19 | loratadine  10mg / capsule  Tablets / capsules | Original drug | Kairuitan | Bayer | Yes (√)  None () | 6Grains (tablets) | 6slice | 17.53 | 2.922 |
|  |  | Anda |  |  | Yes ()  None (√) | 6Grains (tablets) |  |  |  |
| 20 | ibuprofen  200mg / capsule  Tablets / capsules | Original drug | / | / | Yes ()  None (√) | 30Grains (tablets) | / | / | / |
|  |  | Anda |  | Sino US Tianjin Shike Pharmaceutical Co., Ltd | Yes (√)  None () | 30Grains (tablets) | 0.3g * 20 capsules | 16.86 | 0.562 |
| 21 | Hydrochlorothiazide  25mg / capsule  Tablets / capsules | Original drug | Dichlotride | Mershadong | Yes ()  None (√) | 30Grains (tablets) |  |  |  |
|  |  | Anda |  | Changzhou Pharmaceutical Factory Co., Ltd | Yes (√)  None () | 30Grains (tablets) | 100slice | 8.46 | 0.0846 |
| 22 | Azithromycin  250mg / capsule  Tablets / capsules | Original drug | Xi Shumei | Pfizer | Yes (√)  None () | 6Grains (tablets) | 6slice | 59.6 | 9.93 |
|  |  | Anda |  |  | Yes ()  None (√) | 6Grains (tablets) |  |  |  |
| 23 | Amlodipine besylate  5mg / capsule  Tablets / capsules | Original drug | Activating collaterals | Pfizer | Yes (√)  None () | 30Grains (tablets) | 7slice | 29.87 | 4.267 |
|  |  | Anda | Presserda | China Resources SECCO Pharmaceutical Co., Ltd | Yes (√)  None () | 30Grains (tablets) | 14slice | 17.45 | 1.246 |
| 24 | digoxin  25 mg / capsule  Tablets / capsules | Original drug | Lanosine | GlaxoSmithKline | Yes ()  None (√) | 100Grains (tablets) |  |  |  |
|  |  | Anda | Can force | Sanofi (Hangzhou) Pharmaceutical Co., Ltd | Yes (√)  None () | 100Grains (tablets) | 30slice | 30 | 1 |

| Serial number | Common name  Specifications  Dosage form | category | Trade name | Manufacturer | Should I  drugs | Suggestion package  Installation specification | Our company  Installation specification | The package specification price | Minimum order  Bit price |
| --- | --- | --- | --- | --- | --- | --- | --- | --- | --- |
| 25 | tinidazole  500mg / capsule  Tablets / capsules | Original drug | Tindamax | Mission | Yes ()  None (√) | 8Grains (tablets) |  |  |  |
|  |  | Anda |  |  | Yes ()  None (√) | 8Grains (tablets) |  |  |  |
| 26 | Cetirizine hydrochloride  10mg / capsule  Tablets / capsules | Original drug | Xiantemin | UCB pharma | Yes ()  None (√) | 12Grains (tablets) |  |  |  |
|  |  | Anda |  |  | Yes ()  None (√) | 12Grains (tablets) |  |  |  |
| 27 | metronidazole  200mg / capsule  Tablets / capsules | Original drug | Flagyl | Sanofi Aventis | Yes ()  None (√) | 28Grains (tablets) |  |  |  |
|  |  | Anda |  | Huazhong Pharmaceutical Company Limited | Yes (√)  None () | 28Grains (tablets) | 21slice | 1.05 | 0.05 |
| 28 | Nifedipine (sustained release)  20mg / capsule  Tablets / capsules | Original drug | Adalat -retard | Bayer | Yes (√)  None () | 30Grains (tablets) | 30mg * 7 tablets | 26.93 | 3.847 |
|  |  | Anda | Nifuda | Qingdao Huanghai Pharmaceutical Co., Ltd | Yes (√)  None () | 30Grains (tablets) | 30slice | 22.62 | 0.754 |
| 29 | Diphenhydramine hydrochloride  25mg / capsule  Tablets / capsules | Original drug | Benadryl | Johnson | Yes ()  None (√) | 100Grains (tablets) |  |  |  |
|  |  | Anda |  |  | Yes ()  None (√) | 100Grains (tablets) |  |  |  |
| 30 | Doxycycline hydrochloride  100mg / capsule  Tablets / capsules | Original drug | / | / | Yes ()  None (√) | 100Grains (tablets) | / | / | / |
|  |  | Anda | Yongxi | Yongxin Pharmaceutical Industry Co., Ltd | Yes (√)  None () | 100Grains (tablets) | 20grain | 29.23 | 1.4615 |
| 31 | Promethazine hydrochloride  25mg / capsule  Tablets / capsules | Original drug | Phenergan | Sanofi Aventis | Yes ()  None (√) | 20Grains (tablets) |  |  |  |
|  |  | Anda |  |  | Yes ()  None (√) | 20Grains (tablets) |  |  |  |
| 32 | Irbesartan  150mg / capsule  Tablets / capsules | Original drug | Aprovel | Sanofi Aventis | Yes (√)  None () | 7Grains (tablets) | 7slice | 28.56 | 4.08 |
|  |  | Anda | Jiga | Jiangsu Hengrui Pharmaceutical Co., Ltd | Yes (√)  None () | 7Grains (tablets) | 7slice | 6.83 | 0.9757 |

| Serial number | Common name  Specifications  Dosage form | category | Trade name | Manufacturer | Should I  drugs | Suggestion package  Installation specification | Our company  Installation specification | The package specification price | Minimum order  Bit price |
| --- | --- | --- | --- | --- | --- | --- | --- | --- | --- |
| 33 | Losartan potassium  50mg / capsule  Tablets / capsules | Original drug | Kosua | Mershadong | Yes ()  None (√) | 7Grains (tablets) |  |  |  |
|  |  | Anda |  | Yangtze River Pharm | Yes (√)  None () | 7Grains (tablets) | 14slice | 41.74 | 2.9814 |
| 34 | Cefuroxime  250mg / capsule  Tablets / capsules | Original drug | Zinacef | GlaxoSmithKline | Yes (√)  None () | 12Grains (tablets) | 12slice | 32.88 | 2.74 |
|  |  | Anda |  |  | Yes ()  None (√) | 12Grains (tablets) |  |  |  |
| 35 | Enalapril maleate  10mg / capsule  Tablets / capsules | Original drug | Yueningding | Mershadong | Yes ()  None (√) | 30Grains (tablets) |  |  |  |
|  |  | Anda |  | Yangtze River Pharm | Yes (√)  None () | 30Grains (tablets) | 5mg * 16 tablets | 35.12 | 4.39 |
| 36 | Lisinopril  10mg / capsule  Tablets / capsules | Original drug | Jiecirui | AstraZeneca | Yes ()  None (√) | 14Grains (tablets) |  |  |  |
|  |  | Anda |  |  | Yes ()  None (√) | 14Grains (tablets) |  |  |  |
| 37 | Sertraline Hydrochloride  50mg / capsule  Tablets / capsules | Original drug | Zoloft | Pfizer | Yes ()  None (√) | 28Grains (tablets) |  |  |  |
|  |  | Anda | Only he stops | Zhejiang Jingxin Pharmaceutical Co., Ltd | Yes (√)  None () | 28Grains (tablets) | 14slice | 32.92 | 2.3514 |
| 38 | Gliclazide  80mg / capsule  Tablets / capsules | Original drug | Dameikang | servier | Yes (√)  None () | 100Grains (tablets) | 30mg * 30 tablets | 44.17 | 3.926 |
|  |  | Anda |  | Guilin Huaxin Pharmaceutical Co., Ltd | Yes (√)  None () | 100Grains (tablets) | 30mg * 20 capsules | 23.9 | 3.187 |
| 39 | Levofloxacin  500mg / capsule  Tablets / capsules | Original drug | Levaquin | Janssen | Yes ()  None (√) | 6Grains (tablets) |  |  |  |
|  |  | Anda | Cola bituo | No.1 Pharmaceutical Co., Ltd | Yes (√)  None () | 6Grains (tablets) | 4slice | 45.27 | 11.3175 |
| 40 | Chlorphenamine Maleate  4mg / tablet  Tablets / capsules | Original drug | / | / | Yes ()  None (√) | 100Grains (tablets) | / | / | / |
|  |  | Anda |  | Jiangsu Pengyao Pharmaceutical Co., Ltd | Yes (√)  None () | 100Grains (tablets) | 100slice | 5.62 | 0.0562 |

| Serial number | Common name  Specifications  Dosage form | category | Trade name | Manufacturer | Should I  drugs | Suggestion package  Installation specification | Our company  Installation specification | The minimum price of the package specification | Minimum order  Bit price |
| --- | --- | --- | --- | --- | --- | --- | --- | --- | --- |
| 41 | Atorvastatin calcium  20mg / capsule  Tablets / capsules | Original drug | Lipitor | Pfizer | Yes (√)  None () | 7Grains (tablets) | 7slice | 55.49 | 7.9271 |
|  |  | Anda | particularly good | Tianfang Pharmaceutical Co., Ltd | Yes (√)  None () | 7Grains (tablets) | 10mg * 10 capsules | 33.15 | 6.63 |
| 42 | Clomipramine hydrochloride  25mg / capsule  tablet | Original drug | Anafranil | Novartis | Yes ()  None (√) | 50Grains (tablets) |  |  |  |
|  |  | Anda |  |  | Yes ()  None (√) | 50Grains (tablets) |  |  |  |
| 43 | Nimodipine  30mg / capsule  Tablets / capsules | Original drug | nimotop | Bayer | Yes ()  None (√) | 20Grains (tablets) |  |  |  |
|  |  | Anda |  | Zhengda Qingchun Bao Pharmaceutical Co., Ltd | Yes (√)  None () | 20Grains (tablets) | 20mg * 30 tablets | 18 | 0.9 |
| 44 | Clopidogrel bisulfate  75mg / capsule  Tablets / capsules | Original drug | Plavix | Sanofi Aventis | Yes (√)  None () | 7Grains (tablets) | 7slice | 108.25 | 15.4643 |
|  |  | Anda | Taijia | Shenzhen Litai Pharmaceutical Co., Ltd | Yes ()  None (√) | 7Grains (tablets) | 25 mg 20 tablets | 64.8 | 22.68 |
| 45 | Albendazole  200mg / capsule  Tablets / capsules | Original drug | Changchongqing | GlaxoSmithKline | Yes ()  None (√) | 2Grains (tablets) |  |  |  |
|  |  | Anda |  |  | Yes ()  None (√) | 2Grains (tablets) |  |  |  |
| 46 | Propranolol hydrochloride  10mg / capsule  Tablets / capsules | Original drug | Inderal | AstraZeneca | Yes ()  None (√) | 100Grains (tablets) |  |  |  |
|  |  | Anda |  | Jiangsu Yabang Epson Pharmaceutical Co., Ltd | Yes (√)  None () | 100Grains (tablets) | 100slice | 29.8 | 0.298 |
| 47 | erythromycin  250mg / capsule  Tablets / capsules | Original drug | Pantomicina | Abbott | Yes ()  None (√) | 20Grains (tablets) |  |  |  |
|  |  | Anda |  |  | Yes ()  None (√) | 20Grains (tablets) |  |  |  |
| 48 | Mupirocin  2%  Ointment | Original drug | Bactroban | GlaxoSmithKline | Yes (√)  None () | 1Piece / 10g | 1Piece / 10g | 20.18 | 20.18 |
|  |  | Anda |  | Sino US Tianjin Shike Pharmaceutical Co., Ltd | Yes ()  None (√) | 1Piece / 10g |  |  |  |

| Serial number | Common name  Specifications  Dosage form | category | Trade name | Manufacturer | Should I  drugs | Suggestion package  Installation specification | Our company  Installation specification | The package specification price | Minimum order  Bit price |
| --- | --- | --- | --- | --- | --- | --- | --- | --- | --- |
| 49 | Cephalexin  250mg / capsule  Tablets / capsules | Original drug | Keflex | PRAGMA | Yes ()  None (√) | 28Grains (tablets) |  |  |  |
|  |  | Anda |  |  | Yes ()  None (√) | 28Grains (tablets) |  |  |  |
| 50 | Mebendazole  100mg / capsule  Tablets / capsules | Original drug | Vermox | Janssen | Yes (√)  None () | 6Grains (tablets) | 6slice | 2.25 | 0.375 |
|  |  | Anda |  |  | Yes ()  None (√) | 6Grains (tablets) |  |  |  |
