# Supplementary material for: Availability, prices and affordability of essential medicines in Zhejiang Province, China
Source: PLoS One. 2020 Nov 24;15(11):e0241761. doi: 10.1371/journal.pone.0241761 (PMC7685453; doi:10.1371/journal.pone.0241761)
Supplement: S1 File — (ZIP) [file pone.0241761.s001.zip › PLOS ONE Manuscript research data/Research data/Deqing County People's Hospital.docx]

Availability of essential drugs in Zhejiang Province

Note: 1**All blanks**

2. **Package specification**Refers to the total number of packages in a single box, e.g200Press,100Granules (tablets), etc.If there is no recommended package specification, please select the maximum package size of the drug in your company while ensuring that the dosage form and specification remain unchanged.

3. **tablet**It means that the dosage form of the drug can be either tablet or capsule.

4. **Minimum unit price:**For the original research drug, the minimum unit price refers to the minimum unit price of the drug under the determined dosage form, specification, packaging specification, trade name and manufacturer; for generic drugs, the minimum unit price refers to the minimum unit price of the drug under the determined dosage form, specification and packaging specification.

**Usage questionnaire**

| Serial number | Common name  Specifications  Dosage form | category | Trade name | Manufacturer | Should I  drugs | Suggestion package  Installation specification | Our company  Installation specification | The package specification price | Minimum order  Bit price |
| --- | --- | --- | --- | --- | --- | --- | --- | --- | --- |
| 1 | Salbutamol sulfate  100ug / press  Inhaled aerosol | Original drug | ventolin | GlaxoSmithKline | Yes (√)  None () | 200Press (spray) | 200Press (spray) | 20.69 | 0.1034 |
|  |  | Anda |  |  | Yes ()  None (√) | 200Press (spray) |  |  |  |
| 2 | Metformin hydrochloride  500mg / capsule  Tablets / capsules | Original drug | Gehuazhi | Bristol Myers Squibb | Yes (√)  None () | 100Grains (tablets) | 0.5g*20 | 22.77 | 1.1385 |
|  |  | Anda | Andor | It's sunny | Yes (√)  None () | 100Grains (tablets) | 0.5g*60 | 23 | 0.3833 |
| 3 | Bisoprolol fumarate  5mg / capsule  Tablets / capsules | Original drug | Kangke | Merck | Yes (√)  None () | 60Grains (tablets) | 5mg*10 | 28.42 | 2.842 |
|  |  | Anda |  |  | Yes ()  None (√) | 60Grains (tablets) |  |  |  |
| 4 | captopril  25mg / capsule  Tablets / capsules | Original drug | Caputon | Bristol Myers Squibb | Yes (√)  None () | 60Grains (tablets) | 12.5mg*100 | 90.09 |  |
|  |  | Anda |  |  | Yes ()  None (√) | 60Grains (tablets) |  |  |  |
| 5 | Simvastatin  20mg / capsule  Tablets / capsules | Original drug | Shujiangzhi | Mershadong | Yes (√)  None () | 30Grains (tablets) | 20mg*7 | 20.27 | 2.8957 |
|  |  | Anda | Jingbi Shuxin | Zhejiang Jingxin | Yes (√)  None () | 30Grains (tablets) | 20mg*14 | 9.95 | 0.7107 |
| 6 | Amitriptyline hydrochloride  25mg / capsule  Tablets / capsules | Original drug | Tryptizol | Mershadong | Yes (√)  None () | 100Grains (tablets) |  |  |  |
|  |  | Anda |  | Dongting, Hunan | Yes (√)  None () | 100Grains (tablets) | 25mg*100 | 24.8 |  |
| 7 | ciprofloxacin  500mg / capsule  Tablets / capsules | Original drug | Sipple | Bayer | Yes ()  None (√) | 10Grains (tablets) |  |  |  |
|  |  | Anda |  |  | Yes ()  None (√) | 10Grains (tablets) |  |  |  |
| 8 | Compound sulfamethoxazole  8+40mg/ml  Suspension | Original drug | Bactrim | Roche | Yes ()  None (√) | 100ml |  |  |  |
|  |  | Anda |  |  | Yes ()  None (√) | 100ml |  |  |  |

Region: Huzhou City, Zhejiang Province Hospital Name: Deqing people&apos;s Hospital

| Serial number | Common name  Specifications  Dosage form | category | Trade name | Manufacturer | Should I  drugs | Suggestion package  Installation specification | Our company  Installation specification | The package specification price | Minimum order  Bit price |
| --- | --- | --- | --- | --- | --- | --- | --- | --- | --- |
| 9 | Amoxicillin  500mg / capsule  Tablets / capsules | Original drug | Amoxil | GlaxoSmithKline | Yes ()  None (√) | 21Grains (tablets) |  |  |  |
|  |  | Anda |  | Tongda, Shanxi | Yes (√)  None () | 21Grains (tablets) | 0.25g*24 | 20.16 |  |
| 10 | Ceftriaxone sodium  1g / piece  Injections | Original drug | Rocephin | Roche | Yes (√)  None () | 1branch | 1g | 50.94 |  |
|  |  | Anda | Pan Sheng Shu Fu | Taiwan Pan Sheng | Yes (√)  None () | 1branch | 2G | 52.35 |  |
| 11 | omeprazole  20mg / capsule  Tablets / capsules | Original drug | Losec | AstraZeneca | Yes ()  None (√) | 30Grains (tablets) |  |  |  |
|  |  | Anda | Jinaokang | Jinhua Kangenbei | Yes (√)  None () | 30Grains (tablets) | 20mg*14 | 52.7 | 3.7643 |
| 12 | diazepam  5mg / capsule  Tablets / capsules | Original drug | Valium | Roche | Yes ()  None (√) | 100Grains (tablets) |  |  |  |
|  |  | Anda |  | Shandong Xinyi | Yes (√)  None () | 100Grains (tablets) | 2.5mg*20 | 4.9 |  |
| 13 | Oseltamivir  75mg / capsule  Tablets / capsules | Original drug | TMF | Roche | Yes (√)  None () | 100Grains (tablets) | 75mg*10 | 219.31 | 21.931 |
|  |  | Anda | Kewei | Yichang Yangtze River | Yes (√)  None () | 100Grains (tablets) | 75mg*10 | 137.58 | 13.758 |
| 14 | Paracetamol  500mg / capsule  Tablets / capsules | Original drug | Billiton | GlaxoSmithKline | Yes (√)  None () | 10Grains (tablets) | 0.5g*10 | 4.56 | 0.456 |
|  |  | Anda |  |  | Yes (√)  None () | 10Grains (tablets) |  |  |  |
| 15 | diclofenac sodium  25mg / capsule  Tablets / capsules | Original drug | Votalin | Novartis | Yes ()  None (√) | 30Grains (tablets) |  |  |  |
|  |  | Anda | Novotin | Hainan Puli | Yes (√)  None () | 30Grains (tablets) | 0.1g*16 | 20.65 |  |
| 16 | Atenolol  50mg / capsule  Tablets / capsules | Original drug | Tinomin | AstraZeneca | Yes ()  None (√) | 60Grains (tablets) |  |  |  |
|  |  | Anda |  |  | Yes ()  None (√) | 60Grains (tablets) |  |  |  |

| Serial number | Common name  Specifications  Dosage form | category | Trade name | Manufacturer | Should I  drugs | Suggestion package  Installation specification | Our company  Installation specification | The package specification price | Minimum order  Bit price |
| --- | --- | --- | --- | --- | --- | --- | --- | --- | --- |
| 17 | Glimepiride  2mg / capsule  Tablets / capsules | Original drug | Amaryl | Sanofi Aventis | Yes (√)  None () | 15Grains (tablets) | 2mg*15 | 64.31 |  |
|  |  | Anda | Wan Suping | Wanbang, Jiangsu | Yes (√)  None () | 15Grains (tablets) | 1mg*24 | 21.13 |  |
| 18 | Clarithromycin  250mg / capsule  Tablets / capsules | Original drug | Krashen | Abbott | Yes ()  None (√) | 12Grains (tablets) |  |  |  |
|  |  | Anda | Nobond | Jiangsu Hengrui | Yes (√)  None () | 12Grains (tablets) | 0.5g*7 | 27.6 |  |
| 19 | loratadine  10mg / capsule  Tablets / capsules | Original drug | Kairuitan | Bayer | Yes ()  None (√) | 6Grains (tablets) |  |  |  |
|  |  | Anda |  |  | Yes ()  None () | 6Grains (tablets) |  |  |  |
| 20 | ibuprofen  200mg / capsule  Tablets / capsules | Original drug | / | / | Yes ()  None (√) | 30Grains (tablets) | / | / | / |
|  |  | Anda |  | Chifeng Weikang | Yes (√)  None () | 30Grains (tablets) | 0.2g*36 | 22.35 | 0.6208 |
| 21 | Hydrochlorothiazide  25mg / capsule  Tablets / capsules | Original drug | Dichlotride | Mershadong | Yes ()  None () | 30Grains (tablets) |  |  |  |
|  |  | Anda |  | Changzhou pharmaceutical | Yes ()  None () | 30Grains (tablets) | 25mg*100 | 5.01 | 0.0501 |
| 22 | Azithromycin  250mg / capsule  Tablets / capsules | Original drug | Xi Shumei | Pfizer | Yes ()  None () | 6Grains (tablets) |  |  |  |
|  |  | Anda |  | Zhejiang Zhongyi | Yes ()  None () | 6Grains (tablets) | 0.25g*6 | 30.14 | 5.0233 |
| 23 | Amlodipine besylate  5mg / capsule  Tablets / capsules | Original drug | Activating collaterals | Pfizer | Yes ()  None () | 30Grains (tablets) | 5mg*7 | 29.86 | 4.2657 |
|  |  | Anda | Presserda | Beijing SECCO | Yes ()  None () | 30Grains (tablets) | 5mg*14 | 17.3 | 1.2357 |
| 24 | digoxin  25 mg / capsule  Tablets / capsules | Original drug | Lanosine | GlaxoSmithKline | Yes ()  None () | 100Grains (tablets) |  |  |  |
|  |  | Anda | Can force | Sanofi | Yes ()  None () | 100Grains (tablets) | 0.25mg*30 | 30 |  |

| Serial number | Common name  Specifications  Dosage form | category | Trade name | Manufacturer | Should I  drugs | Suggestion package  Installation specification | Our company  Installation specification | The package specification price | Minimum order  Bit price |
| --- | --- | --- | --- | --- | --- | --- | --- | --- | --- |
| 25 | tinidazole  500mg / capsule  Tablets / capsules | Original drug | Tindamax | Mission | Yes ()  None () | 8Grains (tablets) |  |  |  |
|  |  | Anda |  |  | Yes ()  None () | 8Grains (tablets) |  |  |  |
| 26 | Cetirizine hydrochloride  10mg / capsule  Tablets / capsules | Original drug | Xiantemin | UCB pharma | Yes ()  None () | 12Grains (tablets) |  |  |  |
|  |  | Anda |  | Zhejiang hangkang | Yes ()  None () | 12Grains (tablets) | 0.5g*8 | 5.76 |  |
| 27 | metronidazole  200mg / capsule  Tablets / capsules | Original drug | Flagyl | Sanofi Aventis | Yes ()  None () | 28Grains (tablets) |  |  |  |
|  |  | Anda |  |  | Yes ()  None () | 28Grains (tablets) |  |  |  |
| 28 | Nifedipine (sustained release)  20mg / capsule  Tablets / capsules | Original drug | Adalat -retard | Bayer | Yes ()  None () | 30Grains (tablets) |  |  |  |
|  |  | Anda | Nifuda | Qingdao Yellow Sea | Yes ()  None () | 30Grains (tablets) | 20mg*30 | 17.35 | 0.5783 |
| 29 | Diphenhydramine hydrochloride  25mg / capsule  Tablets / capsules | Original drug | Benadryl | Johnson | Yes ()  None () | 100Grains (tablets) |  |  |  |
|  |  | Anda |  |  | Yes ()  None () | 100Grains (tablets) |  |  |  |
| 30 | Doxycycline hydrochloride  100mg / capsule  Tablets / capsules | Original drug | / | / | Yes ()  None () | 100Grains (tablets) | / | / | / |
|  |  | Anda |  |  | Yes ()  None () | 100Grains (tablets) |  |  |  |
| 31 | Promethazine hydrochloride  25mg / capsule  Tablets / capsules | Original drug | Phenergan | Sanofi Aventis | Yes ()  None () | 20Grains (tablets) |  |  |  |
|  |  | Anda |  | Tianjin Lisheng | Yes ()  None () | 20Grains (tablets) | 25mg*100 | 16.6 | 0.166 |
| 32 | Irbesartan  150mg / capsule  Tablets / capsules | Original drug | Aprovel | Sanofi Aventis | Yes ()  None () | 7Grains (tablets) |  |  |  |
|  |  | Anda | Jiga | Jiangsu Hengrui | Yes ()  None () | 7Grains (tablets) | 0.15g*14 | 13.56 | 0.9686 |

| Serial number | Common name  Specifications  Dosage form | category | Trade name | Manufacturer | Should I  drugs | Suggestion package  Installation specification | Our company  Installation specification | The package specification price | Minimum order  Bit price |
| --- | --- | --- | --- | --- | --- | --- | --- | --- | --- |
| 33 | Losartan potassium  50mg / capsule  Tablets / capsules | Original drug | Kosua | Mershadong | Yes ()  None () | 7Grains (tablets) | 0.1g*7 | 48.69 |  |
|  |  | Anda |  | Beijing Wansheng | Yes ()  None () | 7Grains (tablets) | 50mg*14 | 30.7 |  |
| 34 | Cefuroxime  250mg / capsule  Tablets / capsules | Original drug | Zinacef | GlaxoSmithKline | Yes ()  None () | 12Grains (tablets) | 0.25g*12 | 32.59 | 2.7158 |
|  |  | Anda | Dalixin | Shenzhen Zhijun | Yes ()  None () | 12Grains (tablets) | 0.125g*24 | 25.3 |  |
| 35 | Enalapril maleate  10mg / capsule  Tablets / capsules | Original drug | Yueningding | Mershadong | Yes ()  None () | 30Grains (tablets) |  |  |  |
|  |  | Anda |  | Shanghai Hyundai | Yes ()  None () | 30Grains (tablets) | 10mg*16 | 8.5 | 0.5312 |
| 36 | Lisinopril  10mg / capsule  Tablets / capsules | Original drug | Jiecirui | AstraZeneca | Yes ()  None () | 14Grains (tablets) |  |  |  |
|  |  | Anda |  |  | Yes ()  None () | 14Grains (tablets) |  |  |  |
| 37 | Sertraline Hydrochloride  50mg / capsule  Tablets / capsules | Original drug | Zoloft | Pfizer | Yes ()  None () | 28Grains (tablets) | 50mg*14 | 81.72 | 5.8371 |
|  |  | Anda |  | Zhejiang Jingxin | Yes ()  None () | 28Grains (tablets) | 50mg*14 | 43.29 | 3.0921 |
| 38 | Gliclazide  80mg / capsule  Tablets / capsules | Original drug | Dameikang | servier | Yes ()  None () | 100Grains (tablets) |  |  |  |
|  |  | Anda |  | Beijing Wansheng | Yes ()  None () | 100Grains (tablets) | 30mg*60 | 19.84 |  |
| 39 | Levofloxacin  500mg / capsule  Tablets / capsules | Original drug | Levaquin | Janssen | Yes ()  None () | 6Grains (tablets) |  |  |  |
|  |  | Anda | Cola bituo | The first and third party | Yes ()  None () | 6Grains (tablets) | 0.5g*4 | 44.87 | 11.2175 |
| 40 | Chlorphenamine Maleate  4mg / tablet  Tablets / capsules | Original drug | / | / | Yes ()  None () | 100Grains (tablets) | / | / | / |
|  |  | Anda |  | Jiangsu Pengyao | Yes ()  None () | 100Grains (tablets) | 4mg*100 | 9.47 | 0.0947 |

| Serial number | Common name  Specifications  Dosage form | category | Trade name | Manufacturer | Should I  drugs | Suggestion package  Installation specification | Our company  Installation specification | The minimum price of the package specification | Minimum order  Bit price |
| --- | --- | --- | --- | --- | --- | --- | --- | --- | --- |
| 41 | Atorvastatin calcium  20mg / capsule  Tablets / capsules | Original drug | Lipitor | Pfizer | Yes ()  None () | 7Grains (tablets) | 20mg*7 | 55.48 | 7.9257 |
|  |  | Anda | A le | Beijing Jialin | Yes ()  None () | 7Grains (tablets) | 20mg*7 | 39.2 | 5.6000 |
| 42 | Clomipramine hydrochloride  25mg / capsule  tablet | Original drug | Anafranil | Novartis | Yes ()  None () | 50Grains (tablets) |  |  |  |
|  |  | Anda |  | Enhua, Jiangsu | Yes ()  None () | 50Grains (tablets) | 25mg*50 | 15.07 | 0.3014 |
| 43 | Nimodipine  30mg / capsule  Tablets / capsules | Original drug | nimotop | Bayer | Yes ()  None () | 20Grains (tablets) |  |  |  |
|  |  | Anda |  | Zhengda youth treasure | Yes ()  None () | 20Grains (tablets) | 20mg*30 | 18 |  |
| 44 | Clopidogrel bisulfate  75mg / capsule  Tablets / capsules | Original drug | Plavix | Sanofi Aventis | Yes ()  None () | 7Grains (tablets) | 75mg*7 | 108 | 15.4286 |
|  |  | Anda | Taijia | Shenzhen xinlitai | Yes ()  None () | 7Grains (tablets) | 75mg*7 | 58.52 | 8.36 |
| 45 | Albendazole  200mg / capsule  Tablets / capsules | Original drug | Changchongqing | GlaxoSmithKline | Yes ()  None () | 2Grains (tablets) | 0.2g*10 | 12.72 |  |
|  |  | Anda |  |  | Yes ()  None () | 2Grains (tablets) |  |  |  |
| 46 | Propranolol hydrochloride  10mg / capsule  Tablets / capsules | Original drug | Inderal | AstraZeneca | Yes ()  None () | 100Grains (tablets) |  |  |  |
|  |  | Anda |  | Yabang, Jiangsu | Yes (√)  None () | 100Grains (tablets) | 10mg*100 | 29.8 | 0.298 |
| 47 | erythromycin  250mg / capsule  Tablets / capsules | Original drug | Pantomicina | Abbott | Yes ()  None (√) | 20Grains (tablets) |  |  |  |
|  |  | Anda |  |  | Yes ()  None (√) | 20Grains (tablets) |  |  |  |
| 48 | Mupirocin  2%  Ointment | Original drug | Bactroban | GlaxoSmithKline | Yes ()  None (√) | 1Piece / 10g |  |  |  |
|  |  | Anda |  | Ogilvy HK | Yes (√)  None () | 1Piece / 10g | 5g：0.1g | 10.83 | 2.166 |

| Serial number | Common name  Specifications  Dosage form | category | Trade name | Manufacturer | Should I  drugs | Suggestion package  Installation specification | Our company  Installation specification | The package specification price | Minimum order  Bit price |
| --- | --- | --- | --- | --- | --- | --- | --- | --- | --- |
| 49 | Cephalexin  250mg / capsule  Tablets / capsules | Original drug | Keflex | PRAGMA | Yes ()  None (√) | 28Grains (tablets) |  |  |  |
|  |  | Anda |  |  | Yes ()  None (√) | 28Grains (tablets) |  |  |  |
| 50 | Mebendazole  100mg / capsule  Tablets / capsules | Original drug | Vermox | Janssen | Yes ()  None (√) | 6Grains (tablets) |  |  |  |
|  |  | Anda |  |  | Yes ()  None (√) | 6Grains (tablets) |  |  |  |
