# Supplementary material for: Availability, prices and affordability of essential medicines in Zhejiang Province, China
Source: PLoS One. 2020 Nov 24;15(11):e0241761. doi: 10.1371/journal.pone.0241761 (PMC7685453; doi:10.1371/journal.pone.0241761)
Supplement: S1 File — (ZIP) [file pone.0241761.s001.zip › PLOS ONE Manuscript research data/Research data/Feiying Hospital.docx]

The survey of the availability of basic drugs in Zhejiang Province

Note: 1. all should be filled in**All blank spaces**

2. **Packaging specifications**Refers to the total number of packages in a single box, such as200Press,100Grains (tablets), etc.If there is no recommended packing specification, please select the maximum packaging specification of the drug in your company if the dosage form and specification are unchanged.

3. **tablet**It refers to the dosage form of the drug, which can be tablet or capsule.

4. **Minimum unit price:**For the original research drug, the minimum unit price refers to the minimum unit price of the drug under the determined dosage form, specification, packaging specification, commodity name and manufacturer; for generic drugs, the minimum unit price of drugs refers to the minimum unit price of drugs under the determined dosage form, specification and packaging specification.

**Questionnaire on use**

| Serial number | General name  Specifications  Dosage form | category | Trade name | Manufacturer | Is there any right  drugs | Suggested package  Installation specification | Package of the unit  Installation specification | The packing specification price | Minimum single  Price |
| --- | --- | --- | --- | --- | --- | --- | --- | --- | --- |
| 1 | Salbutamol sulfate  100ug/ press  Inhalation aerosol | Original research drug | ventolin | GlaxoSmithKline | Yes ()  None (√) | 200Press (spray) | / | / | / |
|  |  | Anda | / | / | Yes ()  None (√) | 200Press (spray) | / | / | / |
| 2 | Metformin hydrochloride  500mg/ particle  Tablets / capsules | Original research drug | Gehuazhi | Shiguibao | Yes ()  None (√) | 100Granules (pieces) | / | / | / |
|  |  | Anda | Medicon | Shenzhen Zhonglian Pharmaceutical Co., Ltd | Yes (√)  None () | 100Granules (pieces) | 40slice | 10.75 | 0.27 |
| 3 | Bisolol fumarate  5mg/ granule  Tablets / capsules | Original research drug | Kangke | Merck | Yes ()  None (√) | 60Granules (pieces) | / | / | / |
|  |  | Anda | Bosu | Beijing HuaSu Pharmaceutical Co., Ltd | Yes (√)  None () | 60Granules (pieces) | 10slice | 20.78 | 2.08 |
| 4 | captopril  25mg/ particle  Tablets / capsules | Original research drug | Open communication | Shiguibao | Yes ()  None (√) | 60Granules (pieces) | / | / | / |
|  |  | Anda | / | / | Yes ()  None (√) | 60Granules (pieces) | / | / | / |
| 5 | Simvastatin  20mg/ particle  Tablets / capsules | Original research drug | Comfort | Moshadong | Yes (√)  None () | 30Granules (pieces) | 7slice | 20.27 | 2.89 |
|  |  | Anda | Beijing Bishu NEW | Zhejiang Jingxin Pharmaceutical Co., Ltd | Yes (√)  None () | 30Granules (pieces) | 7slice | 4.98 | 0.71 |
| 6 | Amitriptyline hydrochloride  25mg/ particle  Tablets / capsules | Original research drug | Tryptizol | Moshadong | Yes ()  None (√) | 100Granules (pieces) | / | / | / |
|  |  | Anda | / | / | Yes ()  None (√) | 100Granules (pieces) | / | / | / |
| 7 | ciprofloxacin  500mg/ particle  Tablets / capsules | Original research drug | Siple | Bayer | Yes ()  None (√) | 10Granules (pieces) | / | / | / |
|  |  | Anda | / | / | Yes ()  None (√) | 10Granules (pieces) | / | / | / |
| 8 | Compound sulfamethoxazole  8+40mg/ml  Suspension | Original research drug | Bactrim | Roche | Yes ()  None (√) | 100ml | / | / | / |
|  |  | Anda | / | / | Yes ()  None (√) | 100ml | / | / | / |

Region: (Wuxing District) Hospital Name: (Yuehe Feiying community health center))

| Serial number | General name  Specifications  Dosage form | category | Trade name | Manufacturer | Is there any right  drugs | Suggested package  Installation specification | Package of the unit  Installation specification | The packing specification price | Minimum single  Price |
| --- | --- | --- | --- | --- | --- | --- | --- | --- | --- |
| 9 | Amoxicillin  500mg/ particle  Tablets / capsules | Original research drug | Amoxil | GlaxoSmithKline | Yes ()  None (√) | 21Granules (pieces) | / | / | / |
|  |  | Anda | Amoxian | Hong Kong federal Pharmaceutical Factory Co., Ltd | Yes (√)  None () | 21Granules (pieces) | 24grain | 12.9 | 0.54 |
| 10 | Ceftriaxone sodium  1g/ piece  Injection | Original research drug | Rocephin | Roche | Yes ()  None (√) | 1branch | / | / | / |
|  |  | Anda | / | / | Yes ()  None (√) | 1branch | / | / | / |
| 11 | omeprazole  20mg/ particle  Tablets / capsules | Original research drug | Losec | AstraZeneca | Yes (√)  None () | 30Granules (pieces) | 7slice | 41.62 | 5.95 |
|  |  | Anda | Jinokang | Zhejiang Jinhua Kangenbei biopharmaceutical Co., Ltd | Yes (√)  None () | 30Granules (pieces) | 14grain | 52.7 | 3.76 |
| 12 | diazepam  5mg/ granule  Tablets / capsules | Original research drug | Valium | Roche | Yes ()  None (√) | 100Granules (pieces) | / | / | / |
|  |  | Anda | / | / | Yes ()  None (√) | 100Granules (pieces) | / | / | / |
| 13 | Oseltamivir  75mg/ granule  Tablets / capsules | Original research drug | TMF | Roche | Yes ()  None (√) | 100Granules (pieces) | / | / | / |
|  |  | Anda | / | / | Yes (√)  None (√) | 100Granules (pieces) | / | / | / |
| 14 | Paracetamol  500mg/ particle  Tablets / capsules | Original research drug | Must be reasonable | GlaxoSmithKline | Yes ()  None (√) | 10Granules (pieces) | / | / | / |
|  |  | Anda | San Li Tong | Bayer medical and health Co., Ltd | Yes (√)  None () | 10Granules (pieces) | 20grain | 11.28 | 0.564 |
| 15 | diclofenac sodium  25mg/ particle  Tablets / capsules | Original research drug | Butalin | Novartia | Yes ()  None (√) | 30Granules (pieces) | / | / | / |
|  |  | Anda | Antine | China Pharmaceutical University Pharmaceutical Co., Ltd | Yes (√)  None () | 30Granules (pieces) | 20grain | 14.66 | 0.733 |
| 16 | Atenolol  50mg/ particle  Tablets / capsules | Original research drug | Tiannumin | AstraZeneca | Yes ()  None (√) | 60Granules (pieces) | / | / | / |
|  |  | Anda | / | / | Yes ()  None (√) | 60Granules (pieces) | / | / | / |

| Serial number | General name  Specifications  Dosage form | category | Trade name | Manufacturer | Is there any right  drugs | Suggested package  Installation specification | Package of the unit  Installation specification | The packing specification price | Minimum single  Price |
| --- | --- | --- | --- | --- | --- | --- | --- | --- | --- |
| 17 | Glimepiride  2mg/ granule  Tablets / capsules | Original research drug | Amaryl | Sanofi Avant | Yes (√)  None () | 15Granules (pieces) | 15slice | 64.31 | 4.29 |
|  |  | Anda | Wansupin | Jiangsu Wanbang biochemical medicine group Co., Ltd | Yes (√)  None () | 15Granules (pieces) | 30slice | 49.69 | 1.66 |
| 18 | Clarithromycin  250mg / granule  Tablets / capsules | Original research drug | Caraxian | Abbott | Yes ()  None (√) | 12Granules (pieces) |  |  |  |
|  |  | Anda | Norbon | Jiangsu Hengrui Pharmaceutical Co., Ltd | Yes (√)  None () | 12Granules (pieces) | 7slice | 27.6 | 3.94 |
| 19 | loratadine  10mg/ granule  Tablets / capsules | Original research drug | Kairatan | Bayer | Yes ()  None (√) | 6Granules (pieces) | / | / | / |
|  |  | Anda | / | / | Yes ()  None (√) | 6Granules (pieces) | / | / | / |
| 20 | ibuprofen  200mg/ particle  Tablets / capsules | Original research drug | / | / | Yes ()  None (√) | 30Granules (pieces) | / | / | / |
|  |  | Anda | / | / | Yes ()  None (√) | 30Granules (pieces) | / | / | / |
| 21 | Hydrochlorothiazide  25mg/ particle  Tablets / capsules | Original research drug | Dichlotride | Moshadong | Yes ()  None (√) | 30Granules (pieces) | / | / | / |
|  |  | Anda | / | / | Yes ()  None (√) | 30Granules (pieces) | / | / | / |
| 22 | Azithromycin  250mg / granule  Tablets / capsules | Original research drug | Xisummei | Pfizer | Yes ()  None (√) | 6Granules (pieces) | / | / | / |
|  |  | Anda | nothing | Zhejiang China Resources Sanjiu Zhongyi Pharmaceutical Co., Ltd | Yes (√)  None () | 6Granules (pieces) | 6slice | 30.14 | 5.02 |
| 23 | Amlodipine benzenesulfonate  5mg/ granule  Tablets / capsules | Original research drug | Activating collaterals | Pfizer | Yes (√)  None () | 30Granules (pieces) | 7slice | 29.86 | 4.27 |
|  |  | Anda | nothing | Nantong Jiuhe Pharmaceutical Co., Ltd | Yes (√)  None () | 30Granules (pieces) | 30slice | 30.37 | 1.01 |
| 24 | digoxin  0.25mg/ particle  Tablets / capsules | Original research drug | Lanoxin | GlaxoSmithKline | Yes ()  None (√) | 100Granules (pieces) | / | / | / |
|  |  | Anda | / | / | Yes ()  None (√) | 100Granules (pieces) | / | / | / |

| Serial number | General name  Specifications  Dosage form | category | Trade name | Manufacturer | Is there any right  drugs | Suggested package  Installation specification | Package of the unit  Installation specification | The packing specification price | Minimum single  Price |
| --- | --- | --- | --- | --- | --- | --- | --- | --- | --- |
| 25 | tinidazole  500mg/ particle  Tablets / capsules | Original research drug | Tindamax | Mission | Yes ()  None (√) | 8Granules (pieces) | / | / | / |
|  |  | Anda | nothing | Zhejiang hangkang Pharmaceutical Co., Ltd | Yes (√)  None () | 8Granules (pieces) | 8grain | 7.99 | 0.99 |
| 26 | Cetirizine hydrochloride  10mg/ granule  Tablets / capsules | Original research drug | Xiantemin | UCB pharma | Yes ()  None (√) | 12Granules (pieces) | / | / | / |
|  |  | Anda |  |  | Yes ()  None (√) | 12Granules (pieces) | / | / | / |
| 27 | metronidazole  200mg/ particle  Tablets / capsules | Original research drug | Flagyl | Sanofi Avant | Yes ()  None (√) | 28Granules (pieces) | / | / | / |
|  |  | Anda |  |  | Yes ()  None (√) | 28Granules (pieces) | / | / | / |
| 28 | Nifedipine (slow release)  20mg/ particle  Tablets / capsules | Original research drug | Adalat -retard | Bayer | Yes ()  None (√) | 30Granules (pieces) | / | / | / |
|  |  | Anda |  |  | Yes ()  None (√) | 30Granules (pieces) | / | / | / |
| 29 | Pheniramine hydrochloride  25mg/ particle  Tablets / capsules | Original research drug | Benadryl | Johnson | Yes ()  None (√) | 100Granules (pieces) | / | / | / |
|  |  | Anda | / | / | Yes ()  None (√) | 100Granules (pieces) | / | / | / |
| 30 | Doxycycline hydrochloride  100mg/ particle  Tablets / capsules | Original research drug | / | / | Yes ()  None (√) | 100Granules (pieces) | / | / | / |
|  |  | Anda | / | / | Yes ()  None (√) | 100Granules (pieces) | / | / | / |
| 31 | Promethazine hydrochloride  25mg/ particle  Tablets / capsules | Original research drug | Phenergan | Sanofi Avant | Yes ()  None (√) | 20Granules (pieces) | / | / | / |
|  |  | Anda | / | / | Yes ()  None (√) | 20Granules (pieces) | / | / | / |
| 32 | Irbesartan  150mg/ granule  Tablets / capsules | Original research drug | Aprovel | Sanofi Avant | Yes (√)  None () | 7Granules (pieces) | 7slice | 28.5 | 4.07 |
|  |  | Anda | GIGA | Jiangsu Hengrui Pharmaceutical Co., Ltd | Yes (√)  None () | 7Granules (pieces) | 14slice | 13.65 | 0.975 |

| Serial number | General name  Specifications  Dosage form | category | Trade name | Manufacturer | Is there any right  drugs | Suggested package  Installation specification | Package of the unit  Installation specification | The packing specification price | Minimum single  Price |
| --- | --- | --- | --- | --- | --- | --- | --- | --- | --- |
| 33 | Losartan potassium  50mg/ particle  Tablets / capsules | Original research drug | Kosua | Moshadong | Yes (√)  None () | 7Granules (pieces) | 7slice | 48.7 | 6.96 |
|  |  | Anda | nothing | Zhejiang Huahai Pharmaceutical Co., Ltd | Yes (√)  None () | 7Granules (pieces) | 14slice | 57 | 4.07 |
| 34 | Cefuroxime  250mg / granule  Tablets / capsules | Original research drug | Zinacef | GlaxoSmithKline | Yes ()  None (√) | 12Granules (pieces) | / | / | / |
|  |  | Anda | / | / | Yes ()  None (√) | 12Granules (pieces) | / | / | / |
| 35 | Enalapril maleate  10mg/ granule  Tablets / capsules | Original research drug | Yueningding | Moshadong | Yes ()  None (√) | 30Granules (pieces) | / | / | / |
|  |  | Anda | Issu | Jiangsu Pharmaceutical Co., Ltd. of Yangzi River Pharmaceutical Group | Yes (√)  None () | 30Granules (pieces) | 16slice | 21.3 | 1.33 |
| 36 | Renopril  10mg/ granule  Tablets / capsules | Original research drug | Jechery | AstraZeneca | Yes ()  None (√) | 14Granules (pieces) | / | / | / |
|  |  | Anda | / | / | Yes ()  None (√) | 14Granules (pieces) | / | / | / |
| 37 | Sertraline Hydrochloride  50mg/ particle  Tablets / capsules | Original research drug | Zolofev | Pfizer | Yes ()  None (√) | 28Granules (pieces) | / | / | / |
|  |  | Anda | / | / | Yes ()  None (√) | 28Granules (pieces) | / | / | / |
| 38 | Glitzett  80mg/ particle  Tablets / capsules | Original research drug | Damikang | servier | Yes ()  None (√) | 100Granules (pieces) | / | / | / |
|  |  | Anda | / | / | Yes ()  None (√) | 100Granules (pieces) | / | / | / |
| 39 | Levofloxacin  500mg/ particle  Tablets / capsules | Original research drug | Levaquin | Janssen | Yes ()  None (√) | 6Granules (pieces) | / | / | / |
|  |  | Anda | / | / | Yes ()  None (√) | 6Granules (pieces) | / | / | / |
| 40 | Chlorophenamine maleate  4mg/ piece  Tablets / capsules | Original research drug | / | / | Yes ()  None (√) | 100Granules (pieces) | / | / | / |
|  |  | Anda | / | / | Yes ()  None (√) | 100Granules (pieces) | / | / | / |

| Serial number | General name  Specifications  Dosage form | category | Trade name | Manufacturer | Is there any right  drugs | Suggested package  Installation specification | Package of the unit  Installation specification | The minimum price of the packing specification | Minimum single  Price |
| --- | --- | --- | --- | --- | --- | --- | --- | --- | --- |
| 41 | Atorvastatin calcium  20mg/ particle  Tablets / capsules | Original research drug | Lipitor | Pfizer | Yes (√)  None () | 7Granules (pieces) | 77 | 37.28 | 5.33 |
|  |  | Anda | / | / | Yes ()  None (√) | 7Granules (pieces) | / | / | / |
| 42 | Chlormipamine hydrochloride  25mg/ particle  tablet | Original research drug | Anafranil | Novartia | Yes ()  None (√) | 50Granules (pieces) | / | / | / |
|  |  | Anda | / | / | Yes ()  None (√) | 50Granules (pieces) | / | / | / |
| 43 | Nimodipine  30mg/ particle  Tablets / capsules | Original research drug | nimotop | Bayer | Yes (√)  None () | 20Granules (pieces) | 20slice | 27.07 | 1.35 |
|  |  | Anda | / | // | Yes ()  None (√) | 20Granules (pieces) | / | / | / |
| 44 | Clopidogrel bisulfate  75mg/ granule  Tablets / capsules | Original research drug | Plavix | Sanofi Avant | Yes (√)  None () | 7Granules (pieces) | 7grain | 108.19 | 15.45 |
|  |  | Anda | / | / | Yes ()  None (√) | 7Granules (pieces) | / | / | / |
| 45 | Albendazole  200mg/ particle  Tablets / capsules | Original research drug | Enterococcidium | GlaxoSmithKline | Yes ()  None (√) | 2Granules (pieces) | / | / | / |
|  |  | Anda | / | / | Yes ()  None (√) | 2Granules (pieces) | / | / | / |
| 46 | Propranolol hydrochloride  10mg/ granule  Tablets / capsules | Original research drug | Inderal | AstraZeneca | Yes ()  None (√) | 100Granules (pieces) | / | / | / |
|  |  | Anda | / | / | Yes ()  None (√) | 100Granules (pieces) | / | / | / |
| 47 | erythromycin  250mg / granule  Tablets / capsules | Original research drug | Pantomicina | Abbott | Yes ()  None (√) | 20Granules (pieces) | / | / | / |
|  |  | Anda | / | / | Yes ()  None (√) | 20Granules (pieces) | / | / | / |
| 48 | Mopero  2%  Ointment | Original research drug | Bactroban | GlaxoSmithKline | Yes ()  None (√) | 1Branch / 10g | / | / | / |
|  |  | Anda | nothing | Hong Kong Aussie pharmaceutical factory | Yes (√)  None () | 1Branch / 10g | 5g | 10.83 | 2.12 |

| Serial number | General name  Specifications  Dosage form | category | Trade name | Manufacturer | Is there any right  drugs | Suggested package  Installation specification | Package of the unit  Installation specification | The packing specification price | Minimum single  Price |
| --- | --- | --- | --- | --- | --- | --- | --- | --- | --- |
| 49 | Cephalexin  250mg / granule  Tablets / capsules | Original research drug | Keflex | PRAGMA | Yes ()  None (√) | 28Granules (pieces) | / | / | / |
|  |  | Anda | European meaning | Shiyao group Ouyi Pharmaceutical Co., Ltd | Yes (√)  None () | 28Granules (pieces) | 36slice | 16.76 | 0.466 |
| 50 | Mebendazole  100mg/ particle  Tablets / capsules | Original research drug | Vermox | Janssen | Yes ()  None (√) | 6Granules (pieces) | / | / | / |
|  |  | Anda | / | / | Yes ()  None (√) | 6Granules (pieces) | / | / | / |
