# Supplementary material for: Availability, prices and affordability of essential medicines in Zhejiang Province, China
Source: PLoS One. 2020 Nov 24;15(11):e0241761. doi: 10.1371/journal.pone.0241761 (PMC7685453; doi:10.1371/journal.pone.0241761)
Supplement: S1 File — (ZIP) [file pone.0241761.s001.zip › PLOS ONE Manuscript research data/Research data/Mishixiang Street Community Health Service Center, Gongshu District, Hangzhou.docx]

Note: 1**All blanks**

2. **Package specification**Refers to the total number of packages in a single box, e.g200Press,100Granules (tablets), etc.If there is no recommended package specification, please select the maximum package size of the drug in your company while ensuring that the dosage form and specification remain unchanged.

3. **tablet**It means that the dosage form of the drug can be either tablet or capsule.

4. **Minimum unit price:**For the original research drug, the minimum unit price refers to the minimum unit price of the drug under the determined dosage form, specification, packaging specification, trade name and manufacturer; for generic drugs, the minimum unit price refers to the minimum unit price of the drug under the determined dosage form, specification and packaging specification.

**Drug price data collection table**

| Serial number | Common name  Specifications  Dosage form | category | Trade name | Manufacturer | Should I  drugs | Suggestion package  Installation specification | Our company  Installation specification | The package specification price | Minimum order  Bit price |
| --- | --- | --- | --- | --- | --- | --- | --- | --- | --- |
| 1 | Salbutamol sulfate  100ug / press  Inhaled aerosol | Original drug | ventolin | GlaxoSmithKline | Yes (√)  None () | 200Press (spray) | 200Press | 20.69 | 20.69 |
|  |  | Anda |  |  | Yes ()  None (√) | 200Press (spray) |  |  |  |
| 2 | Metformin hydrochloride  500mg / capsule  Tablets / capsules | Original drug | Gehuazhi | Bristol Myers Squibb | Yes (√)  None () | 100Grains (tablets) | 0.5g *20 | 22.97 | 22.97 |
|  |  | Anda |  |  | Yes ()  None (√) | 100Grains (tablets) | 0.25g*36 | 29.81 | 29.81 |
| 3 | Bisoprolol fumarate  5mg / capsule  Tablets / capsules | Original drug | Kangke | Merck | Yes (√)  None () | 60Grains (tablets) | 10 | 28.42 | 28.42 |
|  |  | Anda |  |  | Yes ()  None (√) | 60Grains (tablets) |  |  |  |
| 4 | captopril  25mg / capsule  Tablets / capsules | Original drug | Caputon | Bristol Myers Squibb | Yes (√)  None () | 60Grains (tablets) | 12.5mg 20 | 19.01 | 19.01 |
|  |  | Anda |  |  | Yes ()  None (√) | 60Grains (tablets) |  |  |  |
| 5 | Simvastatin  20mg / capsule  Tablets / capsules | Original drug | Shujiangzhi | Mershadong | Yes (√)  None () | 30Grains (tablets) | 7 | 20.27 | 20.27 |
|  |  | Anda |  |  | Yes (√)  None () | 30Grains (tablets) | 10 | 32.93 | 32.93 |
| 6 | Amitriptyline hydrochloride  25mg / capsule  Tablets / capsules | Original drug | Tryptizol | Mershadong | Yes ()  None (√) | 100Grains (tablets) |  |  |  |
|  |  | Anda |  |  | Yes ()  None (√) | 100Grains (tablets) |  |  |  |
| 7 | ciprofloxacin  500mg / capsule  Tablets / capsules | Original drug | Sipple | Bayer | Yes ()  None (√) | 10Grains (tablets) |  |  |  |
|  |  | Anda |  |  | Yes ()  None () | 10Grains (tablets) |  |  |  |
| 8 | Compound sulfamethoxazole  8+40mg/ml  Suspension | Original drug | Bactrim | Roche | Yes ()  None (√) | 100ml |  |  |  |
|  |  | Anda |  |  | Yes ()  None (√) | 100ml |  |  |  |

| Serial number | Common name  Specifications  Dosage form | category | Trade name | Manufacturer | Should I  drugs | Suggestion package  Installation specification | Our company  Installation specification | The package specification price | Minimum order  Bit price |
| --- | --- | --- | --- | --- | --- | --- | --- | --- | --- |
| 9 | Amoxicillin  500mg / capsule  Tablets / capsules | Original drug | Amoxil | GlaxoSmithKline | Yes ()  None () | 21Grains (tablets) |  |  |  |
|  |  | Anda |  |  | Yes (√)  None () | 21Grains (tablets) | 0.25g 24 | 15.16 | 15.16 |
| 10 | Ceftriaxone sodium  1g / piece  Injections | Original drug | Rocephin | Roche | Yes ()  None (√) | 1branch |  |  |  |
|  |  | Anda |  |  | Yes (√)  None () | 1branch | 2.0g 1 | 53.78 | 53.78 |
| 11 | omeprazole  20mg / capsule  Tablets / capsules | Original drug | Losec | AstraZeneca | Yes ()  None (√) | 30Grains (tablets) |  |  |  |
|  |  | Anda |  |  | Yes (√)  None () | 30Grains (tablets) | 10mg 14 | 42 | 42 |
| 12 | diazepam  5mg / capsule  Tablets / capsules | Original drug | Valium | Roche | Yes ()  None (√) | 100Grains (tablets) |  |  |  |
|  |  | Anda |  |  | Yes ()  None (√) | 100Grains (tablets) |  |  |  |
| 13 | Oseltamivir  75mg / capsule  Tablets / capsules | Original drug | TMF | Roche | Yes ()  None (√) | 100Grains (tablets) |  |  |  |
|  |  | Anda |  |  | Yes ()  None () | 100Grains (tablets) |  |  |  |
| 14 | Paracetamol  500mg / capsule  Tablets / capsules | Original drug | Billiton | GlaxoSmithKline | Yes ()  None (√) | 10Grains (tablets) |  |  |  |
|  |  | Anda |  |  | Yes ()  None (√) | 10Grains (tablets) |  |  |  |
| 15 | diclofenac sodium  25mg / capsule  Tablets / capsules | Original drug | Votalin | Novartis | Yes (√)  None () | 30Grains (tablets) | 75mg 10 | 22.05 | 22.05 |
|  |  | Anda |  |  | Yes ()  None (√) | 30Grains (tablets) |  |  |  |
| 16 | Atenolol  50mg / capsule  Tablets / capsules | Original drug | Tinomin | AstraZeneca | Yes ()  None (√) | 60Grains (tablets) |  |  |  |
|  |  | Anda |  |  | Yes ()  None (√) | 60Grains (tablets) |  |  |  |

| Serial number | Common name  Specifications  Dosage form | category | Trade name | Manufacturer | Should I  drugs | Suggestion package  Installation specification | Our company  Installation specification | The package specification price | Minimum order  Bit price |
| --- | --- | --- | --- | --- | --- | --- | --- | --- | --- |
| 17 | Glimepiride  2mg / capsule  Tablets / capsules | Original drug | Amaryl | Sanofi Aventis | Yes (√)  None () | 15Grains (tablets) | 15 | 64.31 | 64.31 |
|  |  | Anda |  |  | Yes (√)  None () | 15Grains (tablets) | 10 | 23.49 | 23.49 |
| 18 | Clarithromycin  250mg / capsule  Tablets / capsules | Original drug | Krashen | Abbott | Yes ()  None (√) | 12Grains (tablets) |  |  |  |
|  |  | Anda |  |  | Yes (√)  None () | 12Grains (tablets) | 0.5g 3 | 12.6 | 12.6 |
| 19 | loratadine  10mg / capsule  Tablets / capsules | Original drug | Kairuitan | Bayer | Yes ()  None (√) | 6Grains (tablets) |  |  |  |
|  |  | Anda |  |  | Yes (√)  None () | 6Grains (tablets) | 6 | 23.4 | 23.4 |
| 20 | ibuprofen  200mg / capsule  Tablets / capsules | Original drug | / | / | Yes ()  None (√) | 30Grains (tablets) | / | / | / |
|  |  | Anda |  |  | Yes (√)  None () | 30Grains (tablets) | 0.3g 20 | 21.92 | 21.92 |
| 21 | Hydrochlorothiazide  25mg / capsule  Tablets / capsules | Original drug | Dichlotride | Mershadong | Yes ()  None (√) | 30Grains (tablets) |  |  |  |
|  |  | Anda |  |  | Yes (√)  None () | 30Grains (tablets) | 25mg 100 | 8.46 | 8.46 |
| 22 | Azithromycin  250mg / capsule  Tablets / capsules | Original drug | Xi Shumei | Pfizer | Yes ()  None (√) | 6Grains (tablets) |  |  |  |
|  |  | Anda |  |  | Yes (√)  None () | 6Grains (tablets) | 125g (125000 units) 20 | 28.68 | 28.68 |
| 23 | Amlodipine besylate  5mg / capsule  Tablets / capsules | Original drug | Activating collaterals | Pfizer | Yes (√)  None () | 30Grains (tablets) | 5mg 7 | 29.87 | 29.87 |
|  |  | Anda |  |  | Yes (√)  None () | 30Grains (tablets) | 5mg 14 | 39.62 | 39.62 |
| 24 | digoxin  25 mg / capsule  Tablets / capsules | Original drug | Lanosine | GlaxoSmithKline | Yes ()  None (√) | 100Grains (tablets) |  |  |  |
|  |  | Anda |  |  | Yes (√)  None () | 100Grains (tablets) | 0.25mg 30 | 30 | 30 |

| Serial number | Common name  Specifications  Dosage form | category | Trade name | Manufacturer | Yes √ no  drugs | Suggestion package  Installation specification | Our company  Installation specification | The package specification price | Minimum order  Bit price |
| --- | --- | --- | --- | --- | --- | --- | --- | --- | --- |
| 25 | tinidazole  500mg / capsule  Tablets / capsules | Original drug | Tindamax | Mission | Yes ()  None (√) | 8Grains (tablets) |  |  |  |
|  |  | Anda |  |  | Yes (√)  None () | 8Grains (tablets) | 0.5g 8 | 5.76 | 5.76 |
| 26 | Cetirizine hydrochloride  10mg / capsule  Tablets / capsules | Original drug | Xiantemin | UCB pharma | Yes (√)  None () | 12Grains (tablets) | 5 | 16.29 | 16.29 |
|  |  | Anda |  |  | Yes ()  None (√) | 12Grains (tablets) |  |  |  |
| 27 | metronidazole  200mg / capsule  Tablets / capsules | Original drug | Flagyl | Sanofi Aventis | Yes ()  None (√) | 28Grains (tablets) |  |  |  |
|  |  | Anda |  |  | Yes (√)  None () | 28Grains (tablets) | 0.2g 21 | 0.73 | 0.73 |
| 28 | Nifedipine (sustained release)  20mg / capsule  Tablets / capsules | Original drug | Adalat -retard | Bayer | Yes (√)  None () | 30Grains (tablets) | 30mg 7 | 26.93 | 26.93 |
|  |  | Anda |  |  | Yes (√)  None () | 30Grains (tablets) | 20mg 30 | 22.62 | 22.62 |
| 29 | Diphenhydramine hydrochloride  25mg / capsule  Tablets / capsules | Original drug | Benadryl | Johnson | Yes ()  None (√) | 100Grains (tablets) |  |  |  |
|  |  | Anda |  |  | Yes ()  None (√) | 100Grains (tablets) |  |  |  |
| 30 | Doxycycline hydrochloride  100mg / capsule  Tablets / capsules | Original drug | / | / | Yes ()  None (√) | 100Grains (tablets) | / | / | / |
|  |  | Anda |  |  | Yes ()  None (√) | 100Grains (tablets) |  |  |  |
| 31 | Promethazine hydrochloride  25mg / capsule  Tablets / capsules | Original drug | Phenergan | Sanofi Aventis | Yes ()  None (√) | 20Grains (tablets) |  |  |  |
|  |  | Anda |  |  | Yes ()  None (√) | 20Grains (tablets) |  |  |  |
| 32 | Irbesartan  150mg / capsule  Tablets / capsules | Original drug | Aprovel | Sanofi Aventis | Yes (√)  None () | 7Grains (tablets) | 7 | 29.14 | 29.14 |
|  |  | Anda |  |  | Yes (√)  None () | 7Grains (tablets) | 14 | 49.5 | 49.5 |

| Serial number | Common name  Specifications  Dosage form | category | Trade name | Manufacturer | Should I  drugs | Suggestion package  Installation specification | Our company  Installation specification | The package specification price | Minimum order  Bit price |
| --- | --- | --- | --- | --- | --- | --- | --- | --- | --- |
| 33 | Losartan potassium  50mg / capsule  Tablets / capsules | Original drug | Kosua | Mershadong | Yes (√)  None () | 7Grains (tablets) | 14 | 95.04 | 95.04 |
|  |  | Anda |  |  | Yes (√)  None () | 7Grains (tablets) | 7 | 44.14 | 44.14 |
| 34 | Cefuroxime  250mg / capsule  Tablets / capsules | Original drug | Zinacef | GlaxoSmithKline | Yes (√)  None () | 12Grains (tablets) | 12 | 32.88 | 32.88 |
|  |  | Anda |  |  | Yes (√)  None () | 12Grains (tablets) | 0.5g 12 | 41.34 | 41.34 |
| 35 | Enalapril maleate  10mg / capsule  Tablets / capsules | Original drug | Yueningding | Mershadong | Yes ()  None (√) | 30Grains (tablets) |  |  |  |
|  |  | Anda |  |  | Yes ()  None (√) | 30Grains (tablets) |  |  |  |
| 36 | Lisinopril  10mg / capsule  Tablets / capsules | Original drug | Jiecirui | AstraZeneca | Yes ()  None (√) | 14Grains (tablets) |  |  |  |
|  |  | Anda |  |  | Yes ()  None (√) | 14Grains (tablets) |  |  |  |
| 37 | Sertraline Hydrochloride  50mg / capsule  Tablets / capsules | Original drug | Zoloft | Pfizer | Yes (√)  None () | 28Grains (tablets) | 14 | 81.73 | 81.73 |
|  |  | Anda |  |  | Yes ()  None (√) | 28Grains (tablets) |  |  |  |
| 38 | Gliclazide  80mg / capsule  Tablets / capsules | Original drug | Dameikang | servier | Yes (√)  None () | 100Grains (tablets) | 30mg 30 | 44.17 | 44.17 |
|  |  | Anda |  |  | Yes ()  None (√) | 100Grains (tablets) |  |  |  |
| 39 | Levofloxacin  500mg / capsule  Tablets / capsules | Original drug | Levaquin | Janssen | Yes (√)  None () | 6Grains (tablets) | 5 g 4 | 45.27 | 45.27 |
|  |  | Anda |  |  | Yes (√)  None () | 6Grains (tablets) | 0.5g 7 | 21 | 21 |
| 40 | Chlorphenamine Maleate  4mg / tablet  Tablets / capsules | Original drug | / | / | Yes ()  None (√) | 100Grains (tablets) | / | / | / |
|  |  | Anda |  |  | Yes ()  None (√) | 100Grains (tablets) |  |  |  |

District: () Hospital Name: ()

| Serial number | Common name  Specifications  Dosage form | category | Trade name | Manufacturer | Should I  drugs | Suggestion package  Installation specification | Our company  Installation specification | The minimum price of the package specification | Minimum order  Bit price |
| --- | --- | --- | --- | --- | --- | --- | --- | --- | --- |
| 41 | Atorvastatin calcium  20mg / capsule  Tablets / capsules | Original drug | Lipitor | Pfizer | Yes (√)  None () | 7Grains (tablets) | 7 | 55.49 | 55.49 |
|  |  | Anda |  |  | Yes ()  None (√) | 7Grains (tablets) |  |  |  |
| 42 | Clomipramine hydrochloride  25mg / capsule  tablet | Original drug | Anafranil | Novartis | Yes ()  None (√) | 50Grains (tablets) |  |  |  |
|  |  | Anda |  |  | Yes ()  None (√) | 50Grains (tablets) |  |  |  |
| 43 | Nimodipine  30mg / capsule  Tablets / capsules | Original drug | nimotop | Bayer | Yes (√)  None () | 20Grains (tablets) | 20 | 27.07 | 27.07 |
|  |  | Anda |  |  | Yes (√)  None () | 20Grains (tablets) | 20mg 30 | 18 | 18 |
| 44 | Clopidogrel bisulfate  75mg / capsule  Tablets / capsules | Original drug | Plavix | Sanofi Aventis | Yes (√)  None () | 7Grains (tablets) | 7 | 108.25 | 108.25 |
|  |  | Anda |  |  | Yes (√)  None () | 7Grains (tablets) | 25mg 20 | 64.8 | 64.8 |
| 45 | Albendazole  200mg / capsule  Tablets / capsules | Original drug | Changchongqing | GlaxoSmithKline | Yes ()  None (√) | 2Grains (tablets) |  |  |  |
|  |  | Anda |  |  | Yes ()  None (√) | 2Grains (tablets) |  |  |  |
| 46 | Propranolol hydrochloride  10mg / capsule  Tablets / capsules | Original drug | Inderal | AstraZeneca | Yes ()  None (√) | 100Grains (tablets) |  |  |  |
|  |  | Anda |  |  | Yes (√)  None () | 100Grains (tablets) | 10mg 100 | 29.8 | 29.8 |
| 47 | erythromycin  250mg / capsule  Tablets / capsules | Original drug | Pantomicina | Abbott | Yes ()  None (√) | 20Grains (tablets) |  |  |  |
|  |  | Anda |  |  | Yes (√)  None () | 20Grains (tablets) | 20 | 28.6 | 28.6 |
| 48 | Mupirocin  2%  Ointment | Original drug | Bactroban | GlaxoSmithKline | Yes ()  None (√) | 1Piece / 10g |  |  |  |
|  |  | Anda |  |  | Yes (√)  None () | 1Piece / 10g | 5g:0.1g | 10.83 | 10.83 |

| Serial number | Common name  Specifications  Dosage form | category | Trade name | Manufacturer | Should I  drugs | Suggestion package  Installation specification | Our company  Installation specification | The package specification price | Minimum order  Bit price |
| --- | --- | --- | --- | --- | --- | --- | --- | --- | --- |
| 49 | Cephalexin  250mg / capsule  Tablets / capsules | Original drug | Keflex | PRAGMA | Yes ()  None (√) | 28Grains (tablets) |  |  |  |
|  |  | Anda |  |  | Yes ()  None (√) | 28Grains (tablets) |  |  |  |
| 50 | Mebendazole  100mg / capsule  Tablets / capsules | Original drug | Vermox | Janssen | Yes ()  None (√) | 6Grains (tablets) |  |  |  |
|  |  | Anda |  |  | Yes ()  None (√) | 6Grains (tablets) |  |  |  |
