# Supplementary material for: Availability, prices and affordability of essential medicines in Zhejiang Province, China
Source: PLoS One. 2020 Nov 24;15(11):e0241761. doi: 10.1371/journal.pone.0241761 (PMC7685453; doi:10.1371/journal.pone.0241761)
Supplement: S1 File — (ZIP) [file pone.0241761.s001.zip › PLOS ONE Manuscript research data/Research data/Taizhou Jianglin Pharmaceutical Co., Ltd. Pingqiao Store.docx]

**Annex 2 drug price data collection table**

| Serial number | Common name  Specifications  Dosage form | category | Trade name | Manufacturer | Should I  drugs | Suggestion package  Installation specification | Our company  Installation specification | The package specification price | Minimum order  Bit price |
| --- | --- | --- | --- | --- | --- | --- | --- | --- | --- |
| 1 | Salbutamol sulfate  100ug / press  Inhaled aerosol | Original drug | ventolin | GlaxoSmithKline | Yes ()  None (√) | 200Press (spray) |  |  |  |
|  |  | Anda | Li Xinping | Shandong Jingwei | Yes (√)  None () | 200Press (spray) | 200(spray) | 15element | 0.075 |
| 2 | Metformin hydrochloride  500mg / capsule  Tablets / capsules | Original drug | Gehuazhi | Beauty is precious | Yes ()  None (√) | 100Grains (tablets) |  |  |  |
|  |  | Anda |  |  | Yes ()  None (√) | 100Grains (tablets) |  |  |  |
| 3 | Bisoprolol fumarate  5mg / capsule  Tablets / capsules | Original drug | Kangke | Merck | Yes ()  None (√) | 60Grains (tablets) |  |  |  |
|  |  | Anda |  |  | Yes ()  None (√) | 60Grains (tablets) |  |  |  |
| 4 | captopril  25mg / capsule  Tablets / capsules | Original drug | Caputon | Bristol Myers Squibb | Yes ()  None (√) | 60Grains (tablets) |  |  |  |
|  |  | Anda |  |  | Yes ()  None (√) | 60Grains (tablets) |  |  |  |
| 5 | Simvastatin  20mg / capsule  Tablets / capsules | Original drug | Shujiangzhi | Mershadong | Yes ()  None (√) | 30Grains (tablets) |  |  |  |
|  |  | Anda |  |  | Yes ()  None (√) | 30Grains (tablets) |  |  |  |
| 6 | Amitriptyline hydrochloride  25mg / capsule  Tablets / capsules | Original drug | Tryptizol | Mershadong | Yes ()  None (√) | 100Grains (tablets) |  |  |  |
|  |  | Anda |  |  | Yes ()  None (√) | 100Grains (tablets) |  |  |  |
| 7 | ciprofloxacin  500mg / capsule  Tablets / capsules | Original drug | Sipple | Bayer | Yes ()  None (√) | 10Grains (tablets) |  |  |  |
|  |  | Anda |  |  | Yes ()  None (√) | 10Grains (tablets) |  |  |  |
| 8 | Compound sulfamethoxazole  8+40mg/ml  Suspension | Original drug | Bactrim | Roche | Yes ()  None (√) | 100ml |  |  |  |
|  |  | Anda |  |  | Yes ()  None (√) | 100ml |  |  |  |

Area: (Taizhou City) drugstore Name: (Pingqiao store of Taizhou Jianglin pharmaceutical chain Co., Ltd.)

| Serial number | Common name  Specifications  Dosage form | category | Trade name | Manufacturer | Should I  drugs | Suggestion package  Installation specification | Our company  Installation specification | The package specification price | Minimum order  Bit price |
| --- | --- | --- | --- | --- | --- | --- | --- | --- | --- |
| 9 | Amoxicillin  500mg / capsule  Tablets / capsules | Original drug | Amoxil | GlaxoSmithKline | Yes ()  None (√) | 21Grains (tablets) |  |  |  |
|  |  | Anda |  |  | Yes ()  None (√) | 21Grains (tablets) |  |  |  |
| 10 | Ceftriaxone sodium  1g / piece  Injections | Original drug | Rocephin | Roche | Yes ()  None (√) | 1branch |  |  |  |
|  |  | Anda |  |  | Yes ()  None (√) | 1branch |  |  |  |
| 11 | omeprazole  20mg / capsule  Tablets / capsules | Original drug | Losec | AstraZeneca | Yes ()  None (√) | 30Grains (tablets) |  |  |  |
|  |  | Anda |  |  | Yes ()  None (√) | 30Grains (tablets) |  |  |  |
| 12 | diazepam  5mg / capsule  Tablets / capsules | Original drug | Valium | Roche | Yes ()  None (√) | 100Grains (tablets) |  |  |  |
|  |  | Anda |  |  | Yes ()  None (√) | 100Grains (tablets) |  |  |  |
| 13 | Oseltamivir  75mg / capsule  Tablets / capsules | Original drug | TMF | Roche | Yes ()  None (√) | 100Grains (tablets) |  |  |  |
|  |  | Anda |  |  | Yes ()  None (√) | 100Grains (tablets) |  |  |  |
| 14 | Paracetamol  500mg / capsule  Tablets / capsules | Original drug | Billiton | GlaxoSmithKline | Yes ()  None (√) | 10Grains (tablets) |  |  |  |
|  |  | Anda |  |  | Yes ()  None (√) | 10Grains (tablets) |  |  |  |
| 15 | diclofenac sodium  25mg / capsule  Tablets / capsules | Original drug | Votalin | Novartis | Yes ()  None (√) | 30Grains (tablets) |  |  |  |
|  |  | Anda |  |  | Yes ()  None (√) | 30Grains (tablets) |  |  |  |
| 16 | Atenolol  50mg / capsule  Tablets / capsules | Original drug | Tinomin | AstraZeneca | Yes ()  None (√) | 60Grains (tablets) |  |  |  |
|  |  | Anda |  |  | Yes ()  None (√) | 60Grains (tablets) |  |  |  |

| Serial number | Common name  Specifications  Dosage form | category | Trade name | Manufacturer | Should I  drugs | Suggestion package  Installation specification | Our company  Installation specification | The package specification price | Minimum order  Bit price |
| --- | --- | --- | --- | --- | --- | --- | --- | --- | --- |
| 17 | Glimepiride  2mg / capsule  Tablets / capsules | Original drug | Amaryl | Sanofi Aventis | Yes ()  None (√) | 15Grains (tablets) |  |  |  |
|  |  | Anda |  |  | Yes ()  None (√) | 15Grains (tablets) |  |  |  |
| 18 | Clarithromycin  250mg / capsule  Tablets / capsules | Original drug | Krashen | Abbott | Yes ()  None (√) | 12Grains (tablets) |  |  |  |
|  |  | Anda |  |  | Yes ()  None (√) | 12Grains (tablets) |  |  |  |
| 19 | loratadine  10mg / capsule  Tablets / capsules | Original drug | Kairuitan | Bayer | Yes (√)  None () | 6Grains (tablets) | 6grain | 19.5 | 3.25 |
|  |  | Anda |  |  | Yes ()  None (√) | 6Grains (tablets) |  |  |  |
| 20 | ibuprofen  200mg / capsule  Tablets / capsules | Original drug | / | / | Yes ()  None (√) | 30Grains (tablets) | / | / | / |
|  |  | Anda |  |  | Yes ()  None (√) | 30Grains (tablets) |  |  |  |
| 21 | Hydrochlorothiazide  25mg / capsule  Tablets / capsules | Original drug | Dichlotride | Mershadong | Yes ()  None (√) | 30Grains (tablets) |  |  |  |
|  |  | Anda |  |  | Yes ()  None (√) | 30Grains (tablets) |  |  |  |
| 22 | Azithromycin  250mg / capsule  Tablets / capsules | Original drug | Xi Shumei | Pfizer | Yes ()  None (√) | 6Grains (tablets) |  |  |  |
|  |  | Anda |  |  | Yes ()  None (√) | 6Grains (tablets) |  |  |  |
| 23 | Amlodipine besylate  5mg / capsule  Tablets / capsules | Original drug | Activating collaterals | Pfizer | Yes (√)  None () | 30Grains (tablets) | 7grain | 35 | 0.5 |
|  |  | Anda |  |  | Yes ()  None (√) | 30Grains (tablets) |  |  |  |
| 24 | digoxin  25 mg / capsule  Tablets / capsules | Original drug | Lanosine | GlaxoSmithKline | Yes ()  None (√) | 100Grains (tablets) |  |  |  |
|  |  | Anda |  |  | Yes ()  None (√) | 100Grains (tablets) |  |  |  |

| Serial number | Common name  Specifications  Dosage form | category | Trade name | Manufacturer | Should I  drugs | Suggestion package  Installation specification | Our company  Installation specification | The package specification price | Minimum order  Bit price |
| --- | --- | --- | --- | --- | --- | --- | --- | --- | --- |
| 25 | tinidazole  500mg / capsule  Tablets / capsules | Original drug | Tindamax | Mission | Yes ()  None (√) | 8Grains (tablets) |  |  |  |
|  |  | Anda |  |  | Yes ()  None (√) | 8Grains (tablets) |  |  |  |
| 26 | Cetirizine hydrochloride  10mg / capsule  Tablets / capsules | Original drug | Xiantemin | UCB pharma | Yes ()  None (√) | 12Grains (tablets) |  |  |  |
|  |  | Anda |  |  | Yes (√)  None () | 12Grains (tablets) |  |  |  |
| 27 | metronidazole  200mg / capsule  Tablets / capsules | Original drug | Flagyl | Sanofi Aventis | Yes ()  None (√) | 28Grains (tablets) |  |  |  |
|  |  | Anda |  |  | Yes ()  None (√) | 28Grains (tablets) |  |  |  |
| 28 | Nifedipine (sustained release)  20mg / capsule  Tablets / capsules | Original drug | Adalat -retard | Bayer | Yes ()  None (√) | 30Grains (tablets) |  |  |  |
|  |  | Anda |  |  | Yes ()  None (√) | 30Grains (tablets) |  |  |  |
| 29 | Diphenhydramine hydrochloride  25mg / capsule  Tablets / capsules | Original drug | Benadryl | Johnson | Yes ()  None (√) | 100Grains (tablets) |  |  |  |
|  |  | Anda | Lianxin | The Yellow River in Jiangsu Province | Yes (√)  None () | 100Grains (tablets) | 100grain | 1.5 | 0.015 |
| 30 | Doxycycline hydrochloride  100mg / capsule  Tablets / capsules | Original drug | / | / | Yes ()  None (√) | 100Grains (tablets) | / | / | / |
|  |  | Anda |  |  | Yes ()  None (√) | 100Grains (tablets) |  |  |  |
| 31 | Promethazine hydrochloride  25mg / capsule  Tablets / capsules | Original drug | Phenergan | Sanofi Aventis | Yes ()  None (√) | 20Grains (tablets) |  |  |  |
|  |  | Anda |  |  | Yes ()  None (√) | 20Grains (tablets) |  |  |  |
| 32 | Irbesartan  150mg / capsule  Tablets / capsules | Original drug | Aprovel | Sanofi Aventis | Yes ()  None (√) | 7Grains (tablets) |  |  |  |
|  |  | Anda | Jiga | Jiangsu Hengrui | Yes (√)  None () | 7Grains (tablets) | 7grain | 6.83 | 0.975 |

| Serial number | Common name  Specifications  Dosage form | category | Trade name | Manufacturer | Should I  drugs | Suggestion package  Installation specification | Our company  Installation specification | The package specification price | Minimum order  Bit price |
| --- | --- | --- | --- | --- | --- | --- | --- | --- | --- |
| 33 | Losartan potassium  50mg / capsule  Tablets / capsules | Original drug | Kosua | Mershadong | Yes ()  None (√) | 7Grains (tablets) |  |  |  |
|  |  | Anda |  |  | Yes ()  None (√) | 7Grains (tablets) |  |  |  |
| 34 | Cefuroxime  250mg / capsule  Tablets / capsules | Original drug | Zinacef | GlaxoSmithKline | Yes ()  None (√) | 12Grains (tablets) |  |  |  |
|  |  | Anda |  |  | Yes ()  None (√) | 12Grains (tablets) |  |  |  |
| 35 | Enalapril maleate  10mg / capsule  Tablets / capsules | Original drug | Yueningding | Mershadong | Yes ()  None (√) | 30Grains (tablets) |  |  |  |
|  |  | Anda |  |  | Yes ()  None (√) | 30Grains (tablets) |  |  |  |
| 36 | Lisinopril  10mg / capsule  Tablets / capsules | Original drug | Jiecirui | AstraZeneca | Yes ()  None (√) | 14Grains (tablets) |  |  |  |
|  |  | Anda |  |  | Yes ()  None (√) | 14Grains (tablets) |  |  |  |
| 37 | Sertraline Hydrochloride  50mg / capsule  Tablets / capsules | Original drug | Zoloft | Pfizer | Yes ()  None (√) | 28Grains (tablets) |  |  |  |
|  |  | Anda |  |  | Yes ()  None (√) | 28Grains (tablets) |  |  |  |
| 38 | Gliclazide  80mg / capsule  Tablets / capsules | Original drug | Dameikang | servier | Yes ()  None (√) | 100Grains (tablets) |  |  |  |
|  |  | Anda |  |  | Yes ()  None (√) | 100Grains (tablets) |  |  |  |
| 39 | Levofloxacin  500mg / capsule  Tablets / capsules | Original drug | Levaquin | Janssen | Yes ()  None (√) | 6Grains (tablets) |  |  |  |
|  |  | Anda |  |  | Yes ()  None (√) | 6Grains (tablets) |  |  |  |
| 40 | Chlorphenamine Maleate  4mg / tablet  Tablets / capsules | Original drug | / | / | Yes ()  None (√) | 100Grains (tablets) | / | / | / |
|  |  | Anda |  |  | Yes ()  None (√) | 100Grains (tablets) |  |  |  |

| Serial number | Common name  Specifications  Dosage form | category | Trade name | Manufacturer | Should I  drugs | Suggestion package  Installation specification | Our company  Installation specification | The minimum price of the package specification | Minimum order  Bit price |
| --- | --- | --- | --- | --- | --- | --- | --- | --- | --- |
| 41 | Atorvastatin calcium  20mg / capsule  Tablets / capsules | Original drug | Lipitor | Pfizer | Yes ()  None (√) | 7Grains (tablets) |  |  |  |
|  |  | Anda |  |  | Yes ()  None (√) | 7Grains (tablets) |  |  |  |
| 42 | Clomipramine hydrochloride  25mg / capsule  tablet | Original drug | Anafranil | Novartis | Yes ()  None (√) | 50Grains (tablets) |  |  |  |
|  |  | Anda |  |  | Yes ()  None (√) | 50Grains (tablets) |  |  |  |
| 43 | Nimodipine  30mg / capsule  Tablets / capsules | Original drug | nimotop | Bayer | Yes ()  None (√) | 20Grains (tablets) |  |  |  |
|  |  | Anda |  |  | Yes ()  None (√) | 20Grains (tablets) |  |  |  |
| 44 | Clopidogrel hydrochloride  75mg / capsule  Tablets / capsules | Original drug | Plavix | Sanofi Aventis | Yes ()  None (√) | 7Grains (tablets) |  |  |  |
|  |  | Anda |  |  | Yes ()  None (√) | 7Grains (tablets) |  |  |  |
| 45 | Albendazole  200mg / capsule  Tablets / capsules | Original drug | Changchongqing | GlaxoSmithKline | Yes ()  None (√) | 2Grains (tablets) |  |  |  |
|  |  | Anda |  |  | Yes ()  None (√) | 2Grains (tablets) |  |  |  |
| 46 | Propranolol hydrochloride  10mg / capsule  Tablets / capsules | Original drug | Inderal | AstraZeneca | Yes ()  None (√) | 100Grains (tablets) |  |  |  |
|  |  | Anda |  |  | Yes ()  None (√) | 100Grains (tablets) |  |  |  |
| 47 | erythromycin  250mg / capsule  Tablets / capsules | Original drug | Pantomicina | Abbott | Yes ()  None (√) | 20Grains (tablets) |  |  |  |
|  |  | Anda |  |  | Yes ()  None (√) | 20Grains (tablets) |  |  |  |
| 48 | Mupirocin  2%  Ointment | Original drug | Bactroban | GlaxoSmithKline | Yes (√)  None () | 1Piece / 10g |  |  |  |
|  |  | Anda |  |  | Yes ()  None (√) | 1Piece / 10g |  |  |  |

| Serial number | Common name  Specifications  Dosage form | category | Trade name | Manufacturer | Should I  drugs | Suggestion package  Installation specification | Our company  Installation specification | The package specification price | Minimum order  Bit price |
| --- | --- | --- | --- | --- | --- | --- | --- | --- | --- |
| 49 | Cephalexin  250mg / capsule  Tablets / capsules | Original drug | Keflex | PRAGMA | Yes ()  None (√) | 28Grains (tablets) |  |  |  |
|  |  | Anda |  |  | Yes ()  None (√) | 28Grains (tablets) |  |  |  |
| 50 | Mebendazole  100mg / capsule  Tablets / capsules | Original drug | Vermox | Janssen | Yes ()  None (√) | 6Grains (tablets) |  |  |  |
|  |  | Anda |  |  | Yes ()  None (√) | 6Grains (tablets) |  |  |  |
