# Supplementary material for: Availability, prices and affordability of essential medicines in Zhejiang Province, China
Source: PLoS One. 2020 Nov 24;15(11):e0241761. doi: 10.1371/journal.pone.0241761 (PMC7685453; doi:10.1371/journal.pone.0241761)
Supplement: S1 File — (ZIP) [file pone.0241761.s001.zip › PLOS ONE Manuscript research data/Research data/The Second People's Doctor of Yongkang City.docx]

Availability of essential drugs in Zhejiang Province

Note: 1**All blanks**

2. **Package specification**Refers to the total number of packages in a single box, e.g200Press,100Granules (tablets), etc.If there is no recommended package specification, please select the maximum package size of the drug in your company while ensuring that the dosage form and specification remain unchanged.

3. **tablet**It means that the dosage form of the drug can be either tablet or capsule.

4. **Minimum unit price:**For the original research drug, the minimum unit price refers to the minimum unit price of the drug under the determined dosage form, specification, packaging specification, trade name and manufacturer; for generic drugs, the minimum unit price refers to the minimum unit price of the drug under the determined dosage form, specification and packaging specification.

**Usage questionnaire**

| Serial number | Common name  Specifications  Dosage form | category | Trade name | Manufacturer | Should I  drugs | Suggestion package  Installation specification | Our company  Installation specification | The package specification price | Minimum order  Bit price |
| --- | --- | --- | --- | --- | --- | --- | --- | --- | --- |
| 1 | Salbutamol sulfate  100ug / press  Inhaled aerosol | Original drug | ventolin | GlaxoSmithKline | Yes (√)  None () | 200Press (spray) | 200Press (spray) | 20.69 |  |
|  |  | Anda |  |  | Yes ()  None (√) | 200Press (spray) |  |  |  |
| 2 | Metformin hydrochloride  500mg / capsule  Tablets / capsules | Original drug | Gehuazhi | Bristol Myers Squibb | Yes (√)  None () | 100Grains (tablets) | 500mg * 20 tablets | 22.97 |  |
|  |  | Anda | Andor | Zhengda Tianqing Pharmaceutical Group Co., Ltd | Yes (√)  None () | 100Grains (tablets) | 0.5g * 20 tablets | 12 |  |
| 3 | Bisoprolol fumarate  5mg / capsule  Tablets / capsules | Original drug | Kangke | Merck | Yes ()  None (√) | 60Grains (tablets) |  |  |  |
|  |  | Anda |  | Chengdu Yuandong biopharmaceutical Co., Ltd | Yes (√)  None () | 60Grains (tablets) | 5mg * 18 tablets | 39.8 |  |
| 4 | captopril  25mg / capsule  Tablets / capsules | Original drug | Caputon | Bristol Myers Squibb | Yes ()  None (√) | 60Grains (tablets) |  |  |  |
|  |  | Anda |  | Changzhou Pharmaceutical Factory Co., Ltd | Yes (√)  None () | 60Grains (tablets) | 25mg * 100 Tablets | 20.46 |  |
| 5 | Simvastatin  20mg / capsule  Tablets / capsules | Original drug | Shujiangzhi | Mershadong | Yes ()  None (√) | 30Grains (tablets) |  |  |  |
|  |  | Anda |  |  | Yes ()  None (√) | 30Grains (tablets) |  |  |  |
| 6 | Amitriptyline hydrochloride  25mg / capsule  Tablets / capsules | Original drug | Tryptizol | Mershadong | Yes ()  None (√) | 100Grains (tablets) |  |  |  |
|  |  | Anda |  |  | Yes ()  None (√) | 100Grains (tablets) |  |  |  |
| 7 | ciprofloxacin  500mg / capsule  Tablets / capsules | Original drug | Sipple | Bayer | Yes ()  None (√) | 10Grains (tablets) |  |  |  |
|  |  | Anda |  |  | Yes ()  None (√) | 10Grains (tablets) |  |  |  |
| 8 | Compound sulfamethoxazole  8+40mg/ml  Suspension | Original drug | Bactrim | Roche | Yes ()  None (√) | 100ml |  |  |  |
|  |  | Anda |  |  | Yes ()  None (√) | 100ml |  |  |  |

District: Jinhua City Hospital Name: Yongkang Second People's Hospital Yongkang City

| Serial number | Common name  Specifications  Dosage form | category | Trade name | Manufacturer | Should I  drugs | Suggestion package  Installation specification | Our company  Installation specification | The package specification price | Minimum order  Bit price |
| --- | --- | --- | --- | --- | --- | --- | --- | --- | --- |
| 9 | Amoxicillin  500mg / capsule  Tablets / capsules | Original drug | Amoxil | GlaxoSmithKline | Yes ()  None (√) | 21Grains (tablets) |  |  |  |
|  |  | Anda | Amosin | Hong Kong United Pharmaceutical Factory Limited | Yes (√)  None () | 21Grains (tablets) | 0.25g * 24 capsules | 12.9 |  |
| 10 | Ceftriaxone sodium  1g / piece  Injections | Original drug | Rocephin | Roche | Yes ()  None (√) | 1branch |  |  |  |
|  |  | Anda | Pan Sheng Shu Fu | Taiwan Pansheng Pharmaceutical Co., Ltd | Yes (√)  None () | 1branch | 2.0g | 52.35 |  |
| 11 | omeprazole  20mg / capsule  Tablets / capsules | Original drug | Losec | AstraZeneca | Yes ()  None (√) | 30Grains (tablets) |  |  |  |
|  |  | Anda | Jinaokang | Zhejiang Jinhua Kangenbei biopharmaceutical Co., Ltd | Yes (√)  None () | 30Grains (tablets) | 20mg * 14 capsules | 52.7 |  |
| 12 | diazepam  5mg / capsule  Tablets / capsules | Original drug | Valium | Roche | Yes ()  None (√) | 100Grains (tablets) |  |  |  |
|  |  | Anda |  |  | Yes ()  None (√) | 100Grains (tablets) |  |  |  |
| 13 | Oseltamivir  75mg / capsule  Tablets / capsules | Original drug | TMF | Roche | Yes ()  None (√) | 100Grains (tablets) |  |  |  |
|  |  | Anda |  |  | Yes ()  None (√) | 100Grains (tablets) |  |  |  |
| 14 | Paracetamol  500mg / capsule  Tablets / capsules | Original drug | Billiton | GlaxoSmithKline | Yes ()  None (√) | 10Grains (tablets) |  |  |  |
|  |  | Anda |  |  | Yes ()  None (√) | 10Grains (tablets) |  |  |  |
| 15 | diclofenac sodium  25mg / capsule  Tablets / capsules | Original drug | Votalin | Novartis | Yes ()  None (√) | 30Grains (tablets) |  |  |  |
|  |  | Anda | Antine | China Pharmaceutical University Pharmaceutical Co., Ltd | Yes (√)  None () | 30Grains (tablets) | 50mg * 20 capsules | 13.51 |  |
| 16 | Atenolol  50mg / capsule  Tablets / capsules | Original drug | Tinomin | AstraZeneca | Yes ()  None (√) | 60Grains (tablets) |  |  |  |
|  |  | Anda |  |  | Yes ()  None (√) | 60Grains (tablets) |  |  |  |

| Serial number | Common name  Specifications  Dosage form | category | Trade name | Manufacturer | Should I  drugs | Suggestion package  Installation specification | Our company  Installation specification | The package specification price | Minimum order  Bit price |
| --- | --- | --- | --- | --- | --- | --- | --- | --- | --- |
| 17 | Glimepiride  2mg / capsule  Tablets / capsules | Original drug | Amaryl | Sanofi Aventis | Yes (√)  None () | 15Grains (tablets) |  |  |  |
|  |  | Anda | You su | Yangzi River Pharmaceutical Group Guangzhou Hairui Pharmaceutical Co., Ltd | Yes (√)  None () | 15Grains (tablets) | 2mg * 10 tablets | 23.49 |  |
| 18 | Clarithromycin  250mg / capsule  Tablets / capsules | Original drug | Krashen | Abbott | Yes (√)  None () | 12Grains (tablets) |  |  |  |
|  |  | Anda | Nobond | Jiangsu Hengrui Pharmaceutical Co., Ltd | Yes (√)  None () | 12Grains (tablets) | 5g tablets | 27.5 |  |
| 19 | loratadine  10mg / capsule  Tablets / capsules | Original drug | Kairuitan | Bayer | Yes (√)  None () | 6Grains (tablets) | 10mg*6 | 17.53 |  |
|  |  | Anda |  |  | Yes ()  None (√) | 6Grains (tablets) |  |  |  |
| 20 | ibuprofen  200mg / capsule  Tablets / capsules | Original drug | / | / | Yes ()  None (√) | 30Grains (tablets) | / | / | / |
|  |  | Anda |  |  | Yes ()  None (√) | 30Grains (tablets) |  |  |  |
| 21 | Hydrochlorothiazide  25mg / capsule  Tablets / capsules | Original drug | Dichlotride | Mershadong | Yes ()  None (√) | 30Grains (tablets) |  |  |  |
|  |  | Anda |  | Changzhou Pharmaceutical Factory Co., Ltd | Yes (√)  None () | 30Grains (tablets) | 25mg * 100 Tablets | 8.46 |  |
| 22 | Azithromycin  250mg / capsule  Tablets / capsules | Original drug | Xi Shumei | Pfizer | Yes (√)  None () | 6Grains (tablets) | 0.1g * 6 packs | 34.54 |  |
|  |  | Anda |  | Xi&apos;an Daheng Pharmaceutical Co., Ltd | Yes ()  None (√) | 6Grains (tablets) | 0.125g * 20 capsules | 25.5 |  |
| 23 | Amlodipine besylate  5mg / capsule  Tablets / capsules | Original drug | Activating collaterals | Pfizer | Yes (√)  None () | 30Grains (tablets) | 5mg * 7 tablets | 29.86 |  |
|  |  | Anda |  | Nantong Jiuhe Pharmaceutical Co., Ltd | Yes (√)  None () | 30Grains (tablets) | 50mg * 30 tablets | 30.37 |  |
| 24 | digoxin  25 mg / capsule  Tablets / capsules | Original drug | Lanosine | GlaxoSmithKline | Yes ()  None () | 100Grains (tablets) | 0.25mg * 30 tablets | 30 |  |
|  |  | Anda |  |  | Yes ()  None () | 100Grains (tablets) |  |  |  |

| Serial number | Common name  Specifications  Dosage form | category | Trade name | Manufacturer | Should I  drugs | Suggestion package  Installation specification | Our company  Installation specification | The package specification price | Minimum order  Bit price |
| --- | --- | --- | --- | --- | --- | --- | --- | --- | --- |
| 25 | tinidazole  500mg / capsule  Tablets / capsules | Original drug | Tindamax | Mission | Yes ()  None () | 8Grains (tablets) |  |  |  |
|  |  | Anda |  | Zhejiang hangkang Pharmaceutical Co., Ltd | Yes ()  None () | 8Grains (tablets) | 0.5g * 8 tablets | 5.76 |  |
| 26 | Cetirizine hydrochloride  10mg / capsule  Tablets / capsules | Original drug | Xiantemin | UCB pharma | Yes ()  None () | 12Grains (tablets) |  |  |  |
|  |  | Anda |  |  | Yes ()  None () | 12Grains (tablets) |  |  |  |
| 27 | metronidazole  200mg / capsule  Tablets / capsules | Original drug | Flagyl | Sanofi Aventis | Yes ()  None () | 28Grains (tablets) |  |  |  |
|  |  | Anda |  |  | Yes ()  None () | 28Grains (tablets) |  |  |  |
| 28 | Nifedipine (sustained release)  20mg / capsule  Tablets / capsules | Original drug | Adalat -retard | Bayer | Yes ()  None () | 30Grains (tablets) | 30mg * 7 tablets | 26.86 |  |
|  |  | Anda | Na Xintong | Zhejiang talison Pharmaceutical Co., Ltd | Yes ()  None () | 30Grains (tablets) | 20mg * 20 tablets | 15.34 |  |
| 29 | Diphenhydramine hydrochloride  25mg / capsule  Tablets / capsules | Original drug | Benadryl | Johnson | Yes ()  None () | 100Grains (tablets) |  |  |  |
|  |  | Anda |  |  | Yes ()  None () | 100Grains (tablets) |  |  |  |
| 30 | Doxycycline hydrochloride  100mg / capsule  Tablets / capsules | Original drug | / | / | Yes ()  None () | 100Grains (tablets) | / | / | / |
|  |  | Anda |  |  | Yes ()  None () | 100Grains (tablets) |  |  |  |
| 31 | Promethazine hydrochloride  25mg / capsule  Tablets / capsules | Original drug | Phenergan | Sanofi Aventis | Yes ()  None () | 20Grains (tablets) |  |  |  |
|  |  | Anda |  |  | Yes ()  None () | 20Grains (tablets) |  |  |  |
| 32 | Irbesartan  150mg / capsule  Tablets / capsules | Original drug | Aprovel | Sanofi Aventis | Yes ()  None () | 7Grains (tablets) |  |  |  |
|  |  | Anda | Jiga | Jiangsu Hengrui Pharmaceutical Co., Ltd | Yes ()  None () | 7Grains (tablets) | 15 g * 14 tablets | 13.6 |  |

| Serial number | Common name  Specifications  Dosage form | category | Trade name | Manufacturer | Should I  drugs | Suggestion package  Installation specification | Our company  Installation specification | The package specification price | Minimum order  Bit price |
| --- | --- | --- | --- | --- | --- | --- | --- | --- | --- |
| 33 | Losartan potassium  50mg / capsule  Tablets / capsules | Original drug | Kosua | Mershadong | Yes ()  None () | 7Grains (tablets) |  |  |  |
|  |  | Anda |  |  | Yes ()  None () | 7Grains (tablets) |  |  |  |
| 34 | Cefuroxime  250mg / capsule  Tablets / capsules | Original drug | Zinacef | GlaxoSmithKline | Yes ()  None () | 12Grains (tablets) |  |  |  |
|  |  | Anda | Dalixin | Sinopharm group Zhijun (Shenzhen) Pharmaceutical Co., Ltd | Yes ()  None () | 12Grains (tablets) | 0.125g * 24 tablets | 25 |  |
| 35 | Enalapril maleate  10mg / capsule  Tablets / capsules | Original drug | Yueningding | Mershadong | Yes ()  None () | 30Grains (tablets) |  |  |  |
|  |  | Anda |  |  | Yes ()  None () | 30Grains (tablets) |  |  |  |
| 36 | Lisinopril  10mg / capsule  Tablets / capsules | Original drug | Jiecirui | AstraZeneca | Yes ()  None () | 14Grains (tablets) |  |  |  |
|  |  | Anda |  | \| Yichang dongyangguang Changjiang Pharmaceutical Co., Ltd \| \| --- \|     Yichang dongyangguang Changjiang Pharmaceutical Co., Ltd | Yes ()  None () | 14Grains (tablets) | 10mg * 14 tablets | 15.8 |  |
| 37 | Sertraline Hydrochloride  50mg / capsule  Tablets / capsules | Original drug | Zoloft | Pfizer | Yes ()  None () | 28Grains (tablets) |  |  |  |
|  |  | Anda |  |  | Yes ()  None () | 28Grains (tablets) |  |  |  |
| 38 | Gliclazide  80mg / capsule  Tablets / capsules | Original drug | Dameikang | servier | Yes ()  None () | 100Grains (tablets) |  |  |  |
|  |  | Anda |  | Beijing Wansheng Pharmaceutical Co., Ltd | Yes ()  None () | 100Grains (tablets) | 30mg * 60 Tablets | 18.4 |  |
| 39 | Levofloxacin  500mg / capsule  Tablets / capsules | Original drug | Levaquin | Janssen | Yes ()  None () | 6Grains (tablets) |  |  |  |
|  |  | Anda |  | Jiangsu Hengrui Pharmaceutical Co., Ltd | Yes ()  None () | 6Grains (tablets) | 0.5g * 7 tablets | 7.4 |  |
| 40 | Chlorphenamine Maleate  4mg / tablet  Tablets / capsules | Original drug | / | / | Yes ()  None () | 100Grains (tablets) | / | / | / |
|  |  | Anda |  |  | Yes ()  None () | 100Grains (tablets) |  |  |  |

| Serial number | Common name  Specifications  Dosage form | category | Trade name | Manufacturer | Should I  drugs | Suggestion package  Installation specification | Our company  Installation specification | The minimum price of the package specification | Minimum order  Bit price |
| --- | --- | --- | --- | --- | --- | --- | --- | --- | --- |
| 41 | Atorvastatin calcium  20mg / capsule  Tablets / capsules | Original drug | Lipitor | Pfizer | Yes ()  None () | 7Grains (tablets) | 20mg * 7 capsules | 55.48 |  |
|  |  | Anda | particularly good | Tianfang Pharmaceutical Co., Ltd | Yes ()  None () | 7Grains (tablets) | 20mg * 7 capsules | 35.22 |  |
| 42 | Clomipramine hydrochloride  25mg / capsule  tablet | Original drug | Anafranil | Novartis | Yes ()  None () | 50Grains (tablets) |  |  |  |
|  |  | Anda |  |  | Yes ()  None () | 50Grains (tablets) |  |  |  |
| 43 | Nimodipine  30mg / capsule  Tablets / capsules | Original drug | nimotop | Bayer | Yes ()  None () | 20Grains (tablets) |  |  |  |
|  |  | Anda |  |  | Yes ()  None () | 20Grains (tablets) |  |  |  |
| 44 | Clopidogrel bisulfate  75mg / capsule  Tablets / capsules | Original drug | Plavix | Sanofi Aventis | Yes ()  None () | 7Grains (tablets) | 75mg * 7 tablets | 108 |  |
|  |  | Anda |  | Lepu Pharmaceutical Co., Ltd | Yes ()  None () | 7Grains (tablets) | 75mg * 7 tablets | 42.86 |  |
| 45 | Albendazole  200mg / capsule  Tablets / capsules | Original drug | Changchongqing | GlaxoSmithKline | Yes ()  None () | 2Grains (tablets) | 0.2g * 10 tablets | 12.72 |  |
|  |  | Anda |  |  | Yes ()  None () | 2Grains (tablets) |  |  |  |
| 46 | Propranolol hydrochloride  10mg / capsule  Tablets / capsules | Original drug | Inderal | AstraZeneca | Yes ()  None () | 100Grains (tablets) |  |  |  |
|  |  | Anda |  | Jiangsu Yabang Epson Pharmaceutical Co., Ltd | Yes ()  None () | 100Grains (tablets) | 10mg * 100 Tablets | 29.8 |  |
| 47 | erythromycin  250mg / capsule  Tablets / capsules | Original drug | Pantomicina | Abbott | Yes ()  None () | 20Grains (tablets) |  |  |  |
|  |  | Anda | Meihong | Zhejiang China Resources Sanjiu Zhongyi Pharmaceutical Co., Ltd | Yes ()  None () | 20Grains (tablets) | 0.25g * 20 capsules | 27 |  |
| 48 | Mupirocin  2%  Ointment | Original drug | Bactroban | GlaxoSmithKline | Yes ()  None () | 1Piece / 10g |  |  |  |
|  |  | Anda |  |  | Yes ()  None () | 1Piece / 10g |  |  |  |

| Serial number | Common name  Specifications  Dosage form | category | Trade name | Manufacturer | Should I  drugs | Suggestion package  Installation specification | Our company  Installation specification | The package specification price | Minimum order  Bit price |
| --- | --- | --- | --- | --- | --- | --- | --- | --- | --- |
| 49 | Cephalexin  250mg / capsule  Tablets / capsules | Original drug | Keflex | PRAGMA | Yes ()  None () | 28Grains (tablets) |  |  |  |
|  |  | Anda |  |  | Yes ()  None () | 28Grains (tablets) |  |  |  |
| 50 | Mebendazole  100mg / capsule  Tablets / capsules | Original drug | Vermox | Janssen | Yes ()  None () | 6Grains (tablets) |  |  |  |
|  |  | Anda |  |  | Yes ()  None () | 6Grains (tablets) |  |  |  |
