# Supplementary material for: Availability, prices and affordability of essential medicines in Zhejiang Province, China
Source: PLoS One. 2020 Nov 24;15(11):e0241761. doi: 10.1371/journal.pone.0241761 (PMC7685453; doi:10.1371/journal.pone.0241761)
Supplement: S1 File — (ZIP) [file pone.0241761.s001.zip › PLOS ONE Manuscript research data/Research data/The Second People's Hospital of Luqiao District.docx]

Availability of essential drugs in Zhejiang Province

Note: 1**All blanks**

2. **Package specification**Refers to the total number of packages in a single box, e.g200Press,100Granules (tablets), etc.If there is no recommended package specification, please select the maximum package size of the drug in your company while ensuring that the dosage form and specification remain unchanged.

3.**tablet**It means that the dosage form of the drug can be either tablet or capsule.

4.**Minimum unit price:**For the original research drug, the minimum unit price refers to the minimum unit price of the drug under the determined dosage form, specification, packaging specification, trade name and manufacturer; for generic drugs, the minimum unit price refers to the minimum unit price of the drug under the determined dosage form, specification and packaging specification.

**Usage questionnaire**

| Serial number | Common name  Specifications  Dosage form | category | Trade name | Manufacturer | Should I  drugs | Suggestion package  Installation specification | Our company  Installation specification | The package specification price | Minimum order  Bit price |
| --- | --- | --- | --- | --- | --- | --- | --- | --- | --- |
| 1 | Salbutamol sulfate  100ug / press  Inhaled aerosol | Original drug | ventolin | GlaxoSmithKline | Yes (√)  None () | 200Press (spray) | 200Press (spray) | 20.69 | 0.1034 |
|  |  | Anda |  |  | Yes ()  None (√) | 200Press (spray) |  |  |  |
| 2 | Metformin hydrochloride  500mg / capsule  Tablets / capsules | Original drug | Gehuazhi | Bristol Myers Squibb | Yes ()  None (√) | 100Grains (tablets) |  |  |  |
|  |  | Anda | Saintes  Andor | Guizhou shengjitang  Jiangsu Zhengda sunny day | Yes (√)  None () | 100Grains (tablets) | 60 | 39.9 | 0.665 |
| 3 | Bisoprolol fumarate  5mg / capsule  Tablets / capsules | Original drug | Kangke | Merck | Yes (√)  None () | 60Grains (tablets) | 10slice | 28.42 | 2.842 |
|  |  | Anda |  |  | Yes ()  None (√) | 60Grains (tablets) |  |  |  |
| 4 | captopril  25mg / capsule  Tablets / capsules | Original drug | Caputon | Bristol Myers Squibb | Yes ()  None (√) | 60Grains (tablets) |  |  |  |
|  |  | Anda | Yi Jinkang | Zhejiang deende | Yes (√)  None () | 60Grains (tablets) | 100slice | 2.4 | 0.024 |
| 5 | Simvastatin  20mg / capsule  Tablets / capsules | Original drug | Shujiangzhi | Mershadong | Yes ()  None (√) | 30Grains (tablets) |  |  |  |
|  |  | Anda | Simvastatin Dispersible Tablets | Guangzhou Nanxin | Yes (√)  None () | 30Grains (tablets) | 10slice | 32.58 | 32.58 |
| 6 | Amitriptyline hydrochloride  25mg / capsule  Tablets / capsules | Original drug | Tryptizol | Mershadong | Yes ()  None (√) | 100Grains (tablets) |  |  |  |
|  |  | Anda | Amitriptyline tablets | Dongting, Hunan | Yes (√)  None () | 100Grains (tablets) | 100slice | 24.8 | 0.248 |
| 7 | ciprofloxacin  500mg / capsule  Tablets / capsules | Original drug | Sipple | Bayer | Yes ()  None (√) | 10Grains (tablets) |  |  |  |
|  |  | Anda | Ciprofloxacin capsules 250mg | Xinchang, Zhejiang | Yes (√)  None () | 10Grains (tablets) | 10grain | 1.3 | 1.3 |
| 8 | Compound sulfamethoxazole  8+40mg/ml  Suspension | Original drug | Bactrim | Roche | Yes ()  None (√) | 100ml |  |  |  |
|  |  | Anda |  |  | Yes ()  None (√) | 100ml |  |  |  |

Area: (Luqiao District, Taizhou City, Zhejiang Province) Hospital Name: (Luqiao District Second People's Hospital)

| Serial number | Common name  Specifications  Dosage form | category | Trade name | Manufacturer | Should I  drugs | Suggestion package  Installation specification | Our company  Installation specification | The package specification price | Minimum order  Bit price |
| --- | --- | --- | --- | --- | --- | --- | --- | --- | --- |
| 9 | Amoxicillin  500mg / capsule  Tablets / capsules | Original drug | Amoxil | GlaxoSmithKline | Yes ()  None (√) | 21Grains (tablets) |  |  |  |
|  |  | Anda | Amoxicillin Capsules | Hong Kong, Australia and the United States | Yes (√)  None () | 21Grains (tablets) | 20grain | 12.36 | 0.618 |
| 10 | Ceftriaxone sodium  1g / piece  Injections | Original drug | Rocephin | Roche | Yes (√)  None () | 1branch | 1branch | 51.34 | 51.34 |
|  |  | Anda | Ceftriaxone sodium 2G | Taiwan Pan Sheng | Yes (√)  None () | 1branch | 1branch | 53.78 | 53.78 |
| 11 | omeprazole  20mg / capsule  Tablets / capsules | Original drug | Losec | AstraZeneca | Yes (√)  None () | 30Grains (tablets) | 7slice | 74.19 | 10.5986 |
|  |  | Anda | Jinaokang | Conurbay | Yes (√)  None () | 30Grains (tablets) | 14slice | 54.84 | 3.9171 |
| 12 | diazepam  5mg / capsule  Tablets / capsules | Original drug | Valium | Roche | Yes ()  None (√) | 100Grains (tablets) |  |  |  |
|  |  | Anda |  |  | Yes ()  None (√) | 100Grains (tablets) |  |  |  |
| 13 | Oseltamivir  75mg / capsule  Tablets / capsules | Original drug | TMF | Roche | Yes ()  None (√) | 100Grains (tablets) |  |  |  |
|  |  | Anda |  |  | Yes ()  None (√) | 100Grains (tablets) |  |  |  |
| 14 | Paracetamol  500mg / capsule  Tablets / capsules | Original drug | Billiton | GlaxoSmithKline | Yes ()  None (√) | 10Grains (tablets) |  |  |  |
|  |  | Anda |  |  | Yes ()  None (√) | 10Grains (tablets) |  |  |  |
| 15 | diclofenac sodium  25mg / capsule  Tablets / capsules | Original drug | Votalin | Novartis | Yes ()  None (√) | 30Grains (tablets) |  |  |  |
|  |  | Anda |  |  | Yes ()  None (√) | 30Grains (tablets) |  |  |  |
| 16 | Atenolol  50mg / capsule  Tablets / capsules | Original drug | Tinomin | AstraZeneca | Yes ()  None (√) | 60Grains (tablets) |  |  |  |
|  |  | Anda |  |  | Yes ()  None (√) | 60Grains (tablets) |  |  |  |

| Serial number | Common name  Specifications  Dosage form | category | Trade name | Manufacturer | Should I  drugs | Suggestion package  Installation specification | Our company  Installation specification | The package specification price | Minimum order  Bit price |
| --- | --- | --- | --- | --- | --- | --- | --- | --- | --- |
| 17 | Glimepiride  2mg / capsule  Tablets / capsules | Original drug | Amaryl | Sanofi Aventis | Yes (√)  None () | 15Grains (tablets) | 15slice | 64.31 | 64.31 |
|  |  | Anda |  |  | Yes ()  None (√) | 15Grains (tablets) |  |  |  |
| 18 | Clarithromycin  250mg / capsule  Tablets / capsules | Original drug | Krashen | Abbott | Yes ()  None (√) | 12Grains (tablets) |  |  |  |
|  |  | Anda |  |  | Yes ()  None (√) | 12Grains (tablets) |  |  |  |
| 19 | loratadine  10mg / capsule  Tablets / capsules | Original drug | Kairuitan | Bayer | Yes ()  None (√) | 6Grains (tablets) |  |  |  |
|  |  | Anda |  |  | Yes ()  None (√) | 6Grains (tablets) |  |  |  |
| 20 | ibuprofen  200mg / capsule  Tablets / capsules | Original drug | / | / | Yes ()  None (√) | 30Grains (tablets) | / | / | / |
|  |  | Anda |  |  | Yes ()  None (√) | 30Grains (tablets) |  |  |  |
| 21 | Hydrochlorothiazide  25mg / capsule  Tablets / capsules | Original drug | Dichlotride | Mershadong | Yes ()  None (√) | 30Grains (tablets) |  |  |  |
|  |  | Anda | Hydrochlorothiazide | Changzhou Pharm | Yes (√)  None () | 30Grains (tablets) | 100slice | 5.01 | 0.0501 |
| 22 | Azithromycin  250mg / capsule  Tablets / capsules | Original drug | Xi Shumei | Pfizer | Yes ()  None (√) | 6Grains (tablets) |  |  |  |
|  |  | Anda | Azithromycin enteric coated capsules | Zhejiang China Resources Sanjiu | Yes (√)  None () | 6Grains (tablets) | 4grain | 21.24 | 5.3100 |
| 23 | Amlodipine besylate  5mg / capsule  Tablets / capsules | Original drug | Activating collaterals | Pfizer | Yes (√)  None () | 30Grains (tablets) | 7slice | 29.87 | 4.2671 |
|  |  | Anda |  |  | Yes ()  None (√) | 30Grains (tablets) |  |  |  |
| 24 | digoxin  25 mg / capsule  Tablets / capsules | Original drug | Lanosine | GlaxoSmithKline | Yes ()  None (√) | 100Grains (tablets) |  |  |  |
|  |  | Anda | Can force | Hangzhou Sanofi | Yes (√)  None () | 100Grains (tablets) | 30slice | 30.00 | 30.00 |

| Serial number | Common name  Specifications  Dosage form | category | Trade name | Manufacturer | Should I  drugs | Suggestion package  Installation specification | Our company  Installation specification | The package specification price | Minimum order  Bit price |
| --- | --- | --- | --- | --- | --- | --- | --- | --- | --- |
| 25 | tinidazole  500mg / capsule  Tablets / capsules | Original drug | Tindamax | Mission | Yes ()  None (√) | 8Grains (tablets) |  |  |  |
|  |  | Anda |  |  | Yes ()  None (√) | 8Grains (tablets) |  |  |  |
| 26 | Cetirizine hydrochloride  10mg / capsule  Tablets / capsules | Original drug | Xiantemin | UCB pharma | Yes ()  None (√) | 12Grains (tablets) |  |  |  |
|  |  | Anda | Xining | Dongyang, Yichang | Yes (√)  None () | 12Grains (tablets) | 24slice | 25.62 | 1.0675 |
| 27 | metronidazole  200mg / capsule  Tablets / capsules | Original drug | Flagyl | Sanofi Aventis | Yes ()  None (√) | 28Grains (tablets) |  |  |  |
|  |  | Anda | metronidazole tablets | Zhejiang deende | Yes (√)  None () | 28Grains (tablets) | 21slice | 0.73 | 0.0348 |
| 28 | Nifedipine (sustained release)  20mg / capsule  Tablets / capsules | Original drug | Adalat -retard | Bayer | Yes ()  None (√) | 30Grains (tablets) |  |  |  |
|  |  | Anda | Na Xintong | Zhejiang talison | Yes (√)  None () | 30Grains (tablets) | 20slice | 15.34 | 0.767 |
| 29 | Diphenhydramine hydrochloride  25mg / capsule  Tablets / capsules | Original drug | Benadryl | Johnson | Yes ()  None (√) | 100Grains (tablets) |  |  |  |
|  |  | Anda |  |  | Yes ()  None (√) | 100Grains (tablets) |  |  |  |
| 30 | Doxycycline hydrochloride  100mg / capsule  Tablets / capsules | Original drug | / | / | Yes ()  None () | 100Grains (tablets) | / | / | / |
|  |  | Anda | Doxycycline Hydrochloride Tablets | Jiangsu Lianhuan | Yes (√)  None () | 100Grains (tablets) | 100slice | 9.5 | 0.095 |
| 31 | Promethazine hydrochloride  25mg / capsule  Tablets / capsules | Original drug | Phenergan | Sanofi Aventis | Yes ()  None () | 20Grains (tablets) |  |  |  |
|  |  | Anda |  |  | Yes ()  None () | 20Grains (tablets) |  |  |  |
| 32 | Irbesartan  150mg / capsule  Tablets / capsules | Original drug | Aprovel | Sanofi Aventis | Yes (√)  None () | 7Grains (tablets) | 7slice | 28.56 | 4.08 |
|  |  | Anda | Irbesartan dispersible tablets | China Resources double crane benefits people | Yes (√)  None () | 7Grains (tablets) | 12slice | 28.79 | 28.79 |

| Serial number | Common name  Specifications  Dosage form | category | Trade name | Manufacturer | Should I  drugs | Suggestion package  Installation specification | Our company  Installation specification | The package specification price | Minimum order  Bit price |
| --- | --- | --- | --- | --- | --- | --- | --- | --- | --- |
| 33 | Losartan potassium  50mg / capsule  Tablets / capsules | Original drug | Kosua | Mershadong | Yes ()  None (√) | 7Grains (tablets) |  |  |  |
|  |  | Anda | Losartan Potassium Tablets | Zhejiang Huahai | Yes (√)  None () | 7Grains (tablets) | 14slice | 57.00 | 57.00 |
| 34 | Cefuroxime  250mg / capsule  Tablets / capsules | Original drug | Zinacef | GlaxoSmithKline | Yes ()  None (√) | 12Grains (tablets) |  |  |  |
|  |  | Anda |  |  | Yes ()  None (√) | 12Grains (tablets) |  |  |  |
| 35 | Enalapril maleate  10mg / capsule  Tablets / capsules | Original drug | Yueningding | Mershadong | Yes ()  None (√) | 30Grains (tablets) |  |  |  |
|  |  | Anda |  |  | Yes ()  None (√) | 30Grains (tablets) |  |  |  |
| 36 | Lisinopril  10mg / capsule  Tablets / capsules | Original drug | Jiecirui | AstraZeneca | Yes ()  None (√) | 14Grains (tablets) |  |  |  |
|  |  | Anda |  |  | Yes ()  None (√) | 14Grains (tablets) |  |  |  |
| 37 | Sertraline Hydrochloride  50mg / capsule  Tablets / capsules | Original drug | Zoloft | Pfizer | Yes ()  None (√) | 28Grains (tablets) |  |  |  |
|  |  | Anda | Sertraline hydrochloride dispersible tablets | Zhejiang Jingxin | Yes (√)  None () | 28Grains (tablets) | 14slice | 53.64 | 53.64 |
| 38 | Gliclazide  80mg / capsule  Tablets / capsules | Original drug | Dameikang | servier | Yes ()  None (√) | 100Grains (tablets) |  |  |  |
|  |  | Anda |  |  | Yes ()  None (√) | 100Grains (tablets) |  |  |  |
| 39 | Levofloxacin  500mg / capsule  Tablets / capsules | Original drug | Levaquin | Janssen | Yes ()  None (√) | 6Grains (tablets) |  |  |  |
|  |  | Anda | Cola bituo | No.1 Pharmaceutical Co., Ltd | Yes (√)  None () | 6Grains (tablets) | 4slice | 45.27 | 11.3175 |
| 40 | Chlorphenamine Maleate  4mg / tablet  Tablets / capsules | Original drug | / | / | Yes ()  None (√) | 100Grains (tablets) | / | / | / |
|  |  | Anda | Chlorphenamine Maleate Tablets | Jiangsu Pengyao | Yes (√)  None () | 100Grains (tablets) | 100slice | 9.50 | 0.095 |

| Serial number | Common name  Specifications  Dosage form | category | Trade name | Manufacturer | Should I  drugs | Suggestion package  Installation specification | Our company  Installation specification | The minimum price of the package specification | Minimum order  Bit price |
| --- | --- | --- | --- | --- | --- | --- | --- | --- | --- |
| 41 | Atorvastatin calcium  20mg / capsule  Tablets / capsules | Original drug | Lipitor | Pfizer | Yes (√)  None () | 7Grains (tablets) | 7grain | 55.49 | 7.9271 |
|  |  | Anda | particularly good | Henan Tianfang | Yes (√)  None () | 7Grains (tablets) | 7slice | 40.09 | 5.7271 |
| 42 | Clomipramine hydrochloride  25mg / capsule  tablet | Original drug | Anafranil | Novartis | Yes ()  None (√) | 50Grains (tablets) |  |  |  |
|  |  | Anda |  |  | Yes ()  None (√) | 50Grains (tablets) |  |  |  |
| 43 | Nimodipine  30mg / capsule  Tablets / capsules | Original drug | nimotop | Bayer | Yes ()  None (√) | 20Grains (tablets) |  |  |  |
|  |  | Anda |  |  | Yes ()  None (√) | 20Grains (tablets) |  |  |  |
| 44 | Clopidogrel bisulfate  75mg / capsule  Tablets / capsules | Original drug | Plavix | Sanofi Aventis | Yes (√)  None () | 7Grains (tablets) | 7slice | 108.25 | 15.4643 |
|  |  | Anda | Handsome letter | Lepu pharmaceutical | Yes (√)  None () | 7Grains (tablets) | 7slice | 45.49 | 6.4986 |
| 45 | Albendazole  200mg / capsule  Tablets / capsules | Original drug | Changchongqing | GlaxoSmithKline | Yes (√)  None () | 2Grains (tablets) | 10slice | 12.72 | 12.72 |
|  |  | Anda |  |  | Yes ()  None (√) | 2Grains (tablets) |  |  |  |
| 46 | Propranolol hydrochloride  10mg / capsule  Tablets / capsules | Original drug | Inderal | AstraZeneca | Yes ()  None (√) | 100Grains (tablets) |  |  |  |
|  |  | Anda |  |  | Yes ()  None (√) | 100Grains (tablets) |  |  |  |
| 47 | erythromycin  250mg / capsule  Tablets / capsules | Original drug | Pantomicina | Abbott | Yes ()  None (√) | 20Grains (tablets) |  |  |  |
|  |  | Anda |  |  | Yes ()  None (√) | 20Grains (tablets) |  |  |  |
| 48 | Mupirocin  2%  Ointment | Original drug | Bactroban | GlaxoSmithKline | Yes (√)  None () | 1Piece / 10g | 1Piece / 10g | 20.18 | 2.018 |
|  |  | Anda |  |  | Yes ()  None (√) | 1Piece / 10g |  |  |  |

| Serial number | Common name  Specifications  Dosage form | category | Trade name | Manufacturer | Should I  drugs | Suggestion package  Installation specification | Our company  Installation specification | The package specification price | Minimum order  Bit price |
| --- | --- | --- | --- | --- | --- | --- | --- | --- | --- |
| 49 | Cephalexin  250mg / capsule  Tablets / capsules | Original drug | Keflex | PRAGMA | Yes ()  None (√) | 28Grains (tablets) |  |  |  |
|  |  | Anda |  |  | Yes ()  None (√) | 28Grains (tablets) |  |  |  |
| 50 | Mebendazole  100mg / capsule  Tablets / capsules | Original drug | Vermox | Janssen | Yes ()  None (√) | 6Grains (tablets) |  |  |  |
|  |  | Anda |  |  | Yes ()  None (√) | 6Grains (tablets) |  |  |  |
