# Supplementary material for: Availability, prices and affordability of essential medicines in Zhejiang Province, China
Source: PLoS One. 2020 Nov 24;15(11):e0241761. doi: 10.1371/journal.pone.0241761 (PMC7685453; doi:10.1371/journal.pone.0241761)
Supplement: S1 File — (ZIP) [file pone.0241761.s001.zip › PLOS ONE Manuscript research data/Research data/Yinzhou Second Hospital.docx]

Availability of essential drugs in Zhejiang Province

Note: 1**All blanks**

2. **Package specification**Refers to the total number of packages in a single box, e.g200Press,100Granules (tablets), etc.If there is no recommended package specification, please select the maximum package size of the drug in your company while ensuring that the dosage form and specification remain unchanged.

3.**tablet**It means that the dosage form of the drug can be either tablet or capsule.

4.**Minimum unit price:**For the original research drug, the minimum unit price refers to the minimum unit price of the drug under the determined dosage form, specification, packaging specification, trade name and manufacturer; for generic drugs, the minimum unit price refers to the minimum unit price of the drug under the determined dosage form, specification and packaging specification.

**Usage questionnaire**

| Serial number | Common name  Specifications  Dosage form | category | Trade name | Manufacturer | Should I  drugs | Suggestion package  Installation specification | Our company  Installation specification | The package specification price | Minimum order  Bit price |
| --- | --- | --- | --- | --- | --- | --- | --- | --- | --- |
| 1 | Salbutamol sulfate  100ug / press  Inhaled aerosol | Original drug | ventolin | GlaxoSmithKline | Yes ()  None (√) | 200Press (spray) |  |  |  |
|  |  | Anda |  |  | Yes ()  None (√) | 200Press (spray) |  |  |  |
| 2 | Metformin hydrochloride  500mg / capsule  Tablets / capsules | Original drug | Gehuazhi | Bristol Myers Squibb | Yes (√)  None () | 100Grains (tablets) | 20Capsules * 500mg | 22.97 | 1.15 |
|  |  | Anda | Junlida | Beijing Shengyong pharmaceutical | Yes (√)  None () | 100Grains (tablets) | 485 g | 23.79 | 0.5 |
| 3 | Bisoprolol fumarate  5mg / capsule  Tablets / capsules | Original drug | Kangke | Merck | Yes (√)  None () | 60Grains (tablets) | 10Tablets * 5mg | 28.42 | 2.84 |
|  |  | Anda |  |  | Yes ()  None (√) | 60Grains (tablets) |  |  |  |
| 4 | captopril  25mg / capsule  Tablets / capsules | Original drug | Caputon | Bristol Myers Squibb | Yes (√)  None () | 60Grains (tablets) | 100Capsules * 12.5mg | 90.41 | 0.9 |
|  |  | Anda | Captopril Tablets | Zhejiang Asia Pacific | Yes (√)  None () | 60Grains (tablets) | 10025 mg | 1.36 | 0.01 |
| 5 | Simvastatin  20mg / capsule  Tablets / capsules | Original drug | Shujiangzhi | Mershadong | Yes (√)  None () | 30Grains (tablets) | 7Capsules * 20mg | 20.27 | 2.96 |
|  |  | Anda | Simcor | Guangzhou Nanxin | Yes (√)  None () | 30Grains (tablets) | 10Capsules * 20mg | 32.58 | 3.26 |
| 6 | Amitriptyline hydrochloride  25mg / capsule  Tablets / capsules | Original drug | Tryptizol | Mershadong | Yes ()  None (√) | 100Grains (tablets) |  |  |  |
|  |  | Anda |  | Dongting, Hunan | Yes (√)  None () | 100Grains (tablets) | 100Tablet * 25mg | 16.80 | 0.17 |
| 7 | ciprofloxacin  500mg / capsule  Tablets / capsules | Original drug | Sipple | Bayer | Yes ()  None (√) | 10Grains (tablets) |  |  |  |
|  |  | Anda |  |  | Yes ()  None (√) | 10Grains (tablets) |  |  |  |
| 8 | Compound sulfamethoxazole  8+40mg/ml  Suspension | Original drug | Bactrim | Roche | Yes ()  None (√) | 100ml |  |  |  |
|  |  | Anda |  |  | Yes ()  None (√) | 100ml |  |  |  |

Area: (Ningbo) Hospital Name: (the second hospital of Yinzhou District, Ningbo City)

| Serial number | Common name  Specifications  Dosage form | category | Trade name | Manufacturer | Should I  drugs | Suggestion package  Installation specification | Our company  Installation specification | The package specification price | Minimum order  Bit price |
| --- | --- | --- | --- | --- | --- | --- | --- | --- | --- |
| 9 | Amoxicillin  500mg / capsule  Tablets / capsules | Original drug | Amoxil | GlaxoSmithKline | Yes ()  None (√) | 21Grains (tablets) |  |  |  |
|  |  | Anda |  | Zhejiang Kangenbei | Yes (√)  None () | 21Grains (tablets) | 2425 g | 10.10 | 0.40 |
| 10 | Ceftriaxone sodium  1g / piece  Injections | Original drug | Rocephin | Roche | Yes (√)  None () | 1branch | 51.34 * 1 bottle | 51.34 | 51.34 |
|  |  | Anda |  | Taiwan Pansheng pharmaceutical | Yes (√)  None () | 1branch | 53.78 * 1 bottle | 53.78 | 53.78 |
| 11 | omeprazole  20mg / capsule  Tablets / capsules | Original drug | Losec | AstraZeneca | Yes ()  None (√) | 30Grains (tablets) |  |  |  |
|  |  | Anda | Jinaokang | Jinhua Kangenbei | Yes (√)  None () | 30Grains (tablets) | 14Tablets * 10mg | 42 | 3 |
| 12 | diazepam  5mg / capsule  Tablets / capsules | Original drug | Valium | Roche | Yes ()  None (√) | 100Grains (tablets) |  |  |  |
|  |  | Anda |  | Shandong Xinyi | Yes (√)  None () | 100Grains (tablets) | 1005 mg | 9.08 | 0.09 |
| 13 | Oseltamivir  75mg / capsule  Tablets / capsules | Original drug | TMF | Roche | Yes (√)  None () | 100Grains (tablets) | 10Capsules * 75mg | 219.31 | 21.9 |
|  |  | Anda |  |  | Yes ()  None (√) | 100Grains (tablets) |  |  |  |
| 14 | Paracetamol  500mg / capsule  Tablets / capsules | Original drug | Billiton | GlaxoSmithKline | Yes ()  None (√) | 10Grains (tablets) |  |  |  |
|  |  | Anda | Tongli powder | Bayer | Yes (√)  None () | 10Grains (tablets) | 10grain | 5.79 | 0.6 |
| 15 | diclofenac sodium  25mg / capsule  Tablets / capsules | Original drug | Votalin | Novartis | Yes (√)  None () | 30Grains (tablets) | 3025 mg | 16.96 | 0.57 |
|  |  | Anda |  |  | Yes ()  None (√) | 30Grains (tablets) |  |  |  |
| 16 | Atenolol  50mg / capsule  Tablets / capsules | Original drug | Tinomin | AstraZeneca | Yes ()  None (√) | 60Grains (tablets) |  |  |  |
|  |  | Anda |  |  | Yes ()  None (√) | 60Grains (tablets) |  |  |  |

| Serial number | Common name  Specifications  Dosage form | category | Trade name | Manufacturer | Should I  drugs | Suggestion package  Installation specification | Our company  Installation specification | The package specification price | Minimum order  Bit price |
| --- | --- | --- | --- | --- | --- | --- | --- | --- | --- |
| 17 | Glimepiride  2mg / capsule  Tablets / capsules | Original drug | Amaryl | Sanofi Aventis | Yes (√)  None () | 15Grains (tablets) | 15Tablets * 2mg | 64.31 | 4.29 |
|  |  | Anda | You su | Yang Zijiang | Yes (√)  None () | 15Grains (tablets) | 10Tablets * 2mg | 23.49 | 2.35 |
| 18 | Clarithromycin  250mg / capsule  Tablets / capsules | Original drug | Krashen | Abbott | Yes ()  None () | 12Grains (tablets) |  |  |  |
|  |  | Anda | Nobond | Jiangsu Hengrui | Yes (√)  None () | 12Grains (tablets) | 75 g | 29.4 | 4.2 |
| 19 | loratadine  10mg / capsule  Tablets / capsules | Original drug | Kairuitan | Bayer | Yes (√)  None () | 6Grains (tablets) | 6Tablets * 10mg | 17.53 | 2.9 |
|  |  | Anda | Loratadine capsules | Sichuan green leaf Baoguang | Yes (√)  None () | 6Grains (tablets) | 12Tablets * 10mg | 19.99 | 1.67 |
| 20 | ibuprofen  200mg / capsule  Tablets / capsules | Original drug | / | / | Yes ()  None () | 30Grains (tablets) | / | / | / |
|  |  | Anda | Fenbid | Tianjin, China and the United States | Yes (√)  None () | 30Grains (tablets) | 20Granule * 0.3g | 16.86 | 0.843 |
| 21 | Hydrochlorothiazide  25mg / capsule  Tablets / capsules | Original drug | Dichlotride | Mershadong | Yes ()  None () | 30Grains (tablets) |  |  |  |
|  |  | Anda |  | The Place | Yes (√)  None () | 30Grains (tablets) | 10025 mg | 2.13 | 0.02 |
| 22 | Azithromycin  250mg / capsule  Tablets / capsules | Original drug | Xi Shumei | Pfizer | Yes ()  None () | 6Grains (tablets) |  |  |  |
|  |  | Anda |  | Zhejiang Zhongyi | Yes (√)  None () | 6Grains (tablets) | 625 g | 30.82 | 5.15 |
| 23 | Amlodipine besylate  5mg / capsule  Tablets / capsules | Original drug | Activating collaterals | Pfizer | Yes (√)  None () | 30Grains (tablets) | 7Tablets * 5mg | 29.87 | 4.27 |
|  |  | Anda | Shi Huida | Jilin Tianfeng | Yes (√)  None () | 30Grains (tablets) | 75 mg | 13.62 | 1.95 |
| 24 | digoxin  25 mg / capsule  Tablets / capsules | Original drug | Lanosine | GlaxoSmithKline | Yes ()  None (√) | 100Grains (tablets) |  |  |  |
|  |  | Anda |  | Shanghai Xinyi | Yes (√)  None () | 100Grains (tablets) | 3025 g | 30 | 1.0 |

| Serial number | Common name  Specifications  Dosage form | category | Trade name | Manufacturer | Should I  drugs | Suggestion package  Installation specification | Our company  Installation specification | The package specification price | Minimum order  Bit price |
| --- | --- | --- | --- | --- | --- | --- | --- | --- | --- |
| 25 | tinidazole  500mg / capsule  Tablets / capsules | Original drug | Tindamax | Mission | Yes ()  None (√) | 8Grains (tablets) |  |  |  |
|  |  | Anda |  |  | Yes ()  None (√) | 8Grains (tablets) |  |  |  |
| 26 | Cetirizine hydrochloride  10mg / capsule  Tablets / capsules | Original drug | Xiantemin | UCB pharma | Yes ()  None (√) | 12Grains (tablets) |  |  |  |
|  |  | Anda | Cetirizine Hydrochloride Tablets | Shandong lunanbeite | Yes (√)  None () | 12Grains (tablets) | 24Tablets * 10mg | 15.86 | 0.66 |
| 27 | metronidazole  200mg / capsule  Tablets / capsules | Original drug | Flagyl | Sanofi Aventis | Yes ()  None (√) | 28Grains (tablets) |  |  |  |
|  |  | Anda | metronidazole tablets | Zhejiang deende | Yes (√)  None () | 28Grains (tablets) | 21Tablet * 0.2g | 0.74 | 0.04 |
| 28 | Nifedipine (sustained release)  20mg / capsule  Tablets / capsules | Original drug | Adalat -retard | Bayer | Yes (√)  None () | 30Grains (tablets) | 7Tablets * 30mg | 26.93 | 3.85 |
|  |  | Anda | extended release nifedipine tablets | Honglin, Beijing | Yes (√)  None () | 30Grains (tablets) | 14Tablets * 30mg | 41.90 | 3.00 |
| 29 | Diphenhydramine hydrochloride  25mg / capsule  Tablets / capsules | Original drug | Benadryl | Johnson | Yes ()  None (√) | 100Grains (tablets) |  |  |  |
|  |  | Anda |  |  | Yes ()  None (√) | 100Grains (tablets) |  |  |  |
| 30 | Doxycycline hydrochloride  100mg / capsule  Tablets / capsules | Original drug | / | / | Yes ()  None () | 100Grains (tablets) | / | / | / |
|  |  | Anda | Doxycycline hydrochloride | Jiangsu Lianhuan Pharmaceutical Co., Ltd | Yes (√)  None () | 100Grains (tablets) | 100Capsules * 0.1g | 9.50 | 0.095 |
| 31 | Promethazine hydrochloride  25mg / capsule  Tablets / capsules | Original drug | Phenergan | Sanofi Aventis | Yes ()  None (√) | 20Grains (tablets) |  |  |  |
|  |  | Anda |  |  | Yes ()  None (√) | 20Grains (tablets) |  |  |  |
| 32 | Irbesartan  150mg / capsule  Tablets / capsules | Original drug | Aprovel | Sanofi Aventis | Yes (√)  None () | 7Grains (tablets) | 715 g | 28.56 | 4.08 |
|  |  | Anda | Kosu | Yangtze River in Jiangsu Province | Yes (√)  None () | 7Grains (tablets) | 12Capsules * 75mg | 10.40 | 0.87 |

| Serial number | Common name  Specifications  Dosage form | category | Trade name | Manufacturer | Should I  drugs | Suggestion package  Installation specification | Our company  Installation specification | The package specification price | Minimum order  Bit price |
| --- | --- | --- | --- | --- | --- | --- | --- | --- | --- |
| 33 | Losartan potassium  50mg / capsule  Tablets / capsules | Original drug | Kosua | Mershadong | Yes (√)  None () | 7Grains (tablets) | 7Capsules * 0.1g | 48.74 | 6.96 |
|  |  | Anda | Losartan potassium capsule | Beijing Wansheng | Yes (√)  None () | 7Grains (tablets) | 14Tablets * 50mg | 30.99 | 2.2 |
| 34 | Cefuroxime  250mg / capsule  Tablets / capsules | Original drug | Zinacef | GlaxoSmithKline | Yes (√)  None () | 12Grains (tablets) | 24Capsules * 0.125g | 28.94 | 1.21 |
|  |  | Anda |  |  | Yes ()  None (√) | 12Grains (tablets) |  |  |  |
| 35 | Enalapril maleate  10mg / capsule  Tablets / capsules | Original drug | Yueningding | Mershadong | Yes ()  None (√) | 30Grains (tablets) |  |  |  |
|  |  | Anda | Yisu | Yangtze River in Jiangsu Province | Yes (√)  None () | 30Grains (tablets) | 16Tablet * 5mg | 16 | 1 |
| 36 | Lisinopril  10mg / capsule  Tablets / capsules | Original drug | Jiecirui | AstraZeneca | Yes ()  None (√) | 14Grains (tablets) |  |  |  |
|  |  | Anda | Lisinopril Tabelets | Yichang Yangtze River | Yes (√)  None () | 14Grains (tablets) | 14Tablets * 10mg | 16.24 | 1.16 |
| 37 | Sertraline Hydrochloride  50mg / capsule  Tablets / capsules | Original drug | Zoloft | Pfizer | Yes ()  None (√) | 28Grains (tablets) |  |  |  |
|  |  | Anda | Sertraline hydrochloride tablets | Huahai pharmaceutical | Yes (√)  None () | 28Grains (tablets) | 14Tablet * 50mg | 51.46 | 0.0014 |
| 38 | Gliclazide  80mg / capsule  Tablets / capsules | Original drug | Dameikang | servier | Yes (√)  None () | 100Grains (tablets) | 30Tablet * 60mg | 75.53 | 1.26 |
|  |  | Anda | Gliclazide Sustained Release Capsules | Hangzhou Guoguang | Yes (√)  None () | 100Grains (tablets) | 30Tablets * 30mg | 35.98 | 1.20 |
| 39 | Levofloxacin  500mg / capsule  Tablets / capsules | Original drug | Levaquin | Janssen | Yes ()  None (√) | 6Grains (tablets) |  |  |  |
|  |  | Anda | Laili Postcard | Xinchang Pharmaceutical Factory | Yes (√)  None () | 6Grains (tablets) | 12Granules * 0.2g | 12.67 | 1.06 |
| 40 | Chlorphenamine Maleate  4mg / tablet  Tablets / capsules | Original drug | / | / | Yes ()  None () | 100Grains (tablets) | / | / | / |
|  |  | Anda | Chlorphenamine Maleate Tablets | Jiangsu Pengyao | Yes (√)  None () | 100Grains (tablets) | 100Tablets * 4mg | 7.31 | 0.07 |

| Serial number | Common name  Specifications  Dosage form | category | Trade name | Manufacturer | Should I  drugs | Suggestion package  Installation specification | Our company  Installation specification | The minimum price of the package specification | Minimum order  Bit price |
| --- | --- | --- | --- | --- | --- | --- | --- | --- | --- |
| 41 | Atorvastatin calcium  20mg / capsule  Tablets / capsules | Original drug | Lipitor | Pfizer | Yes (√)  None () | 7Grains (tablets) | 7Capsules * 20mg | 55.49 | 7.93 |
|  |  | Anda | Atorvastatin calcium capsules | Topfond Pharmaceutical | Yes (√)  None () | 7Grains (tablets) | 7Capsules * 20mg | 40.09 | 5.73 |
| 42 | Clomipramine hydrochloride  25mg / capsule  tablet | Original drug | Anafranil | Novartis | Yes ()  None (√) | 50Grains (tablets) |  |  |  |
|  |  | Anda |  |  | Yes ()  None (√) | 50Grains (tablets) |  |  |  |
| 43 | Nimodipine  30mg / capsule  Tablets / capsules | Original drug | nimotop | Bayer | Yes ()  None (√) | 20Grains (tablets) |  |  |  |
|  |  | Anda | Nimodipine tablets | Zhengda youth treasure | Yes (√)  None () | 20Grains (tablets) | 30Capsules * 20mg | 18 | 0.6 |
| 44 | Clopidogrel bisulfate  75mg / capsule  Tablets / capsules | Original drug | Plavix | Sanofi Aventis | Yes (√)  None () | 7Grains (tablets) | 7Capsules * 75mg | 108.25 | 15.46 |
|  |  | Anda | Clopidogrel Sulfate Tablets | Lepu pharmaceutical | Yes (√)  None () | 7Grains (tablets) | 10Capsules * 75mg | 64.38 | 6.44 |
| 45 | Albendazole  200mg / capsule  Tablets / capsules | Original drug | Changchongqing | GlaxoSmithKline | Yes (√)  None () | 2Grains (tablets) | 10Granules * 0.2g | 12.72 | 1.27 |
|  |  | Anda |  |  | Yes ()  None (√) | 2Grains (tablets) |  |  |  |
| 46 | Propranolol hydrochloride  10mg / capsule  Tablets / capsules | Original drug | Inderal | AstraZeneca | Yes ()  None (√) | 100Grains (tablets) |  |  |  |
|  |  | Anda |  |  | Yes ()  None (√) | 100Grains (tablets) |  |  |  |
| 47 | erythromycin  250mg / capsule  Tablets / capsules | Original drug | Pantomicina | Abbott | Yes ()  None (√) | 20Grains (tablets) |  |  |  |
|  |  | Anda | Erythromycin enteric capsules | Zhejiang Zhongyi | Yes (√)  None () | 20Grains (tablets) | 2025 g | 28.60 | 1.43 |
| 48 | Mupirocin  2%  Ointment | Original drug | Bactroban | GlaxoSmithKline | Yes (√)  None () | 1Piece / 10g | 1Piece / 10g | 20.18 | 20.18 |
|  |  | Anda |  |  | Yes ()  None (√) | 1Piece / 10g |  |  |  |

| Serial number | Common name  Specifications  Dosage form | category | Trade name | Manufacturer | Should I  drugs | Suggestion package  Installation specification | Our company  Installation specification | The package specification price | Minimum order  Bit price |
| --- | --- | --- | --- | --- | --- | --- | --- | --- | --- |
| 49 | Cephalexin  250mg / capsule  Tablets / capsules | Original drug | Keflex | PRAGMA | Yes ()  None (√) | 28Grains (tablets) |  |  |  |
|  |  | Anda |  |  | Yes ()  None (√) | 28Grains (tablets) |  |  |  |
| 50 | Mebendazole  100mg / capsule  Tablets / capsules | Original drug | Vermox | Janssen | Yes ()  None (√) | 6Grains (tablets) |  |  |  |
|  |  | Anda |  |  | Yes ()  None (√) | 6Grains (tablets) |  |  |  |
