# Supplementary material for: Availability, prices and affordability of essential medicines in Zhejiang Province, China
Source: PLoS One. 2020 Nov 24;15(11):e0241761. doi: 10.1371/journal.pone.0241761 (PMC7685453; doi:10.1371/journal.pone.0241761)
Supplement: Attachment — Submitted filename: Response to Reviewers.docx [file pone.0241761.s002.docx]

**Rebuttal letter**

Dear Susan Horton
Thanks you very much for your comments and suggestions.

As suggested, We have made amendments to the academic papers, in accordance with the requirements of the academic editors, and responded to the comments made by the reviewers. The details are attached below. Some of our basic research data is written in Chinese, which may be difficult to understand.

We have polished English and revised academic papers.We have revised the manuscript, according to the comments and suggestions of reviewers and editor, and responded, point by point to, the comments as listed below. Some of the references we cited are published in domestic academic journals in China and may not be found in foreign databases.

I would like to re-submit this revised manuscript to PLOS ONE, and hope it is acceptable for publication in the journal.

Looking forward to hearing from you soon.

With kindest regards,

Yours Sincerely

QiuCheng Tao

Replies to Reviewers and Editor

First of all, we thank both reviewers and editor for their positive and constructive comments and suggestions.

Replies to Reviewer #1:

1. Overall the article need language improvement by an expert; thus I will not comment on the matters related to language and grammar.

We have polished English and revised academic papers.

2. Abstract - Conclusions - affordability is one of the main objs but it was not mentioned.

Objective: To evaluate the availability, prices and availability of essential medicines in Zhejiang Province, China.

3. Methods section - you mentioned 'an additional 5 cities' but you only mentioned 4+1 - it is confusing - 5 or 6 cities in total?

A total of 6 cities, 1 provincial capital city plus 5 cities

Hangzhou, Ningbo, Shaoxing, Huzhou, Taizhou,Jiaxing.

4. Methods section - what does 'scale' mean in this context ?

The scale represents that the hospital we selected has a good level, and relevant data can be obtained

5. many researchers used the WHO/HAI methodology but why this methodology? any strengths/advantages? please explain.

In terms of the results of many academic papers, this method is the best.This method can intuitively analyze the drug availability and affordability of the survey site.

6. Figure 1 - check the spelling for Omeprazole; and the use of cap vs small letter

already edited

7. Table 7 - font type - be consistent with the main text; check the use of cap vs small letters for drug name

already edited

8. Discussion - does this province use diff policy and regulation from other provinces in China? good for the International readers to understand

The "4 + 7" policy is a national policy, which not only affects hospitals, pharmaceutical manufacturers, and pharmacies across the country, but also has its own policies among various provinces. The policies of Zhejiang Province are different from those of other provinces in China. The policies of Zhejiang Province encourage public and private pharmacies to increase the availability of medicines and reduce the affordability of medicines.
9. Discussion - lack of studies from other countries that are relevant; were not included in the discussion

Our research is based on the actual situation of Zhejiang Province, China, and is different from other countries. Other countries do not have the same policies as China, so the research results are also different.

10. Conclusion - sentence 1 - 'inadequate' - what does this clearly mean? shortages? not available?

"Inadequate" means that there is a shortage of medicines and pharmacies cannot supply medicines

11. Include recommendations section at the end of the Discussion section - move the ones mentioned in the Conclusion section.

Has been modified

12. Include the strength, limitations and study implications in the last part of the Discussion section

In this study, we adopted a systematic research method to study related issues, combined with the actual situation and policies to study the availability and affordability of drugs in public and private pharmacies in Zhejiang.

This study has several limitations. First of all, we use the research method based on WHO / HAI, which may have some errors from the real situation in Zhejiang Province. A better method may need to be developed in the future. Second, the relevant data is only applicable to the date of data collection and may not reflect the average availability over a period of time. Finally, this study analyzes the price components of essential medicines in China using the standardized WHO / HAI method. However, only some medicines are included in the analysis. Due to confidentiality reasons, some data have errors and missing.

The availability and affordability of medicines is a complex systemic problem.The question first needs to clarify the essential causes of the shortage of drugs, on this basis, grasp the key factors, and propose solutions from the aspects of drug prices, market environment, policy systems and legislation. The smooth implementation of these solutions also requires the coordination and cooperation of various government departments and stakeholders of the drug supply. Only when the main bodies of the drug supply chain work together can the current issues of great concern be solved to the greatest extent.

Replies to Reviewer #2:

1. It’s not clear why Tables 1 and 2 are presented separately, since the availabilities in Table 3 and the price summaries in Tables 4 and 5 appear to be based on the combined data.

Table 1 and Table 2 are listed separately for space reasons, and their meanings are the same. The availability and affordability of OB and LPG representing public and private pharmacies

2. Tables 1 and 2 at times use “0” and other times “-“ in the columns. Initially I assumed that “-“ meant not applicable and that these items were excluded from Table 4 but that appears not to be the case, and probably “0” and “-“ actually mean the same thing.

0 means the drug was not obtained in the pharmacy

3. Table 4 has a note below the table stating this applies to 37 medicines (although the superscript “a” does not appear in the table, so I am guessing here). There is no reason given for this, since there are more than 37 LPG drugs available in that sector according to Tables 1 and 2.

In fact, the meaning of Table 4 is that indicates that generic products in the public sector were generally sold at more than 5 times of the international reference prices. “A Thirty-seven medicines included.” Is a clerical error.

4. Similarly, in Table 5 has a line in the table this time indicating that the comparisons 22 and 36 medicines, however this does not seem to equate to the number of cases where the drug was available in the private sector. Some explanation would be desirable.

The average of 22 and 36 drugs represented by random sampling in public and private pharmacies

5. I have an overall concern with the WHO-HAI methodology. The importance of non-availability of the “lowest priced generic” requires careful definition. Is the “lowest priced generic” defined for China (or for Zhejiang province) and if so how? How much difference is there between the prices of the lowest priced generic and the second lowest priced generic for these drugs? What is the policy significance of the lowest-priced one being unavailable in a circumstance where possibility the second lowest-priced one is available, and how does that compare to a situation where the lowest-priced one is considerably less expensive than the next lowest-price one? It is possible that when the WHO originated the methodology back in 2003 fewer generics were available, so this wasn’t as much of an issue as it perhaps is now.

Lowest price generic equivalents are defined as the same product sold under the generic name with the lowest unit price at each medicine outlet at the time of data collection in the survey.

6. How did the authors take account of the fact that the international reference prices were for 2015, while the data were collected in 2018. Do international pharmaceutical prices not change much over three years?

Our research project started in 2018 and it takes a lot of time, manpower and material resources to collect data. The international reference price for 2015 was chosen because it was the latest reference data when the project started.

7. Given that the policy issues are the main area in which this paper distinguishes itself from scores (or hundreds) of similar studies, it would be worth getting an English-first-language health expert to read the discussion and conclusion sections and make the policy implications clearer for example the term “subsidies” in the Discussion section sometimes seems to mean government contributions to the hospital budget rather than a subsidy per se; and “The essence of “4 + 7” is to exchange price by quantity” would be better explained “…is to use bulk purchases to drive down prices” or something similar.
Language has been polished

Replies to the Editors note and suggestions:

1. Please ensure that your manuscript meets PLOS ONE's style requirements, including those for file naming.

Has been modified

2.  Please address the following:
- Please ensure you have thoroughly discussed any potential limitations of this study within the Discussion section.
- Please ensure your methods have been described in sufficient detail so that these analyses could be performed again. In the text you state "Data collection also uses the WHO/HAI methodology", which is not sufficient.

Has been modified

3. We noticed you have some minor occurrence of overlapping text with the following previous publication(s), which needs to be addressed:

Some of the references we cited are published in domestic academic journals in China and may not be found in foreign databases.

4. In your Data Availability statement, you have not specified where the minimal data set underlying the results described in your manuscript can be found. PLOS defines a study's minimal data set as the underlying data used to reach the conclusions drawn in the manuscript and any additional data required to replicate the reported study findings in their entirety. All PLOS journals require that the minimal data set be made fully available. For more information about our data policy, please see

Due to commercial secrecy, the data we collected cannot be fully disclosed, and we have processed it in the system.

5. Thank you for stating the following financial disclosure:

'NO - Include this sentence at the end of your statement: The funders had no role in study design, data collection and analysis, decision to publish, or preparation of the manuscript.'

At this time, please address the following queries:

a. Please clarify the sources of funding (financial or material support) for your study. List the grants or organizations that supported your study, including funding received from your institution.
b. State what role the funders took in the study. If the funders had no role in your study, please state: “The funders had no role in study design, data collection and analysis, decision to publish, or preparation of the manuscript.”
c. If any authors received a salary from any of your funders, please state which authors and which funders.
d. If you did not receive any funding for this study, please state: “The authors received no specific funding for this work.”

Please include your amended statements within your cover letter; we will change the online submission form on your behalf.

The authors received no specific funding for this work.”

6. Please amend the manuscript submission data (via Edit Submission) to include author Bobo Yan.

Has been modified

7. Please amend either the abstract on the online submission form (via Edit Submission) or the abstract in the manuscript so that they are identical.

Has been modified

8. Please ensure that you refer to Figure 1 in your text as, if accepted, production will need this reference to link the reader to the figure.

Has been modified

9. We note you have included tables to which you do not refer in the text of your manuscript. Please ensure that you refer to Tables 2, 5 and 7 in your text; if accepted, production will need this reference to link the reader to the Tables.

Has been modified

10. Please include captions for your Supporting Information files at the end of your manuscript, and update any in-text citations to match accordingly. Please see our Supporting Information guidelines for more

Has been modified
