# Supplementary material for: Availability, prices and affordability of essential medicines in Zhejiang Province, China
Source: PLoS One. 2020 Nov 24;15(11):e0241761. doi: 10.1371/journal.pone.0241761 (PMC7685453; doi:10.1371/journal.pone.0241761)
Supplement: Attachment — Submitted filename: Response to Reviewers.docx [file pone.0241761.s003.docx]

**Rebuttal letter**

Dear Susan Horton
Thanks you very much for your comments and suggestions.

As suggested, We have made amendments to the academic papers, in accordance with the requirements of the academic editors, and responded to the comments made by the reviewers. The details are attached below. Some of our basic research data is written in Chinese, which may be difficult to understand. Our research data has been uploaded to the submission system. Because it is the most primitive data, it is all in Chinese。

We have revised the manuscript, according to the comments and suggestions of reviewers and editor, and responded, point by point to, the comments as listed below. Some of the references we cited are published in domestic academic journals in China and may not be found in foreign databases.

I would like to re-submit this revised manuscript to PLOS ONE, and hope it is acceptable for publication in the journal.

Looking forward to hearing from you soon.

With kindest regards.

Yours Sincerely

Guojun Sun

Replies to Reviewers and Editor

First of all, we thank both reviewers and editor for their positive and constructive comments and suggestions.

Replies to Reviewer #1:

1. Please see REviewer 1's comments below. I also provided responses as Reviewer 2, which I include here.This paper is somewhat improved in methodology following revisions, although the quality of the written English remains an issue. A big disadvantage is that the authors did not provide a “track changes” version of the manuscript and thus it was hard to see whether the changes really had been implemented as requested. I would request that in the next revision a track changes version be provided.

In the "track changes" version, we marked the modified part in red font, so that the reviewers can see our modification more clearly

2. One issue for me is that “Lowest Priced Generic” was not clearly defined. Does this mean that the authors identified a lowest priced generic brand overall (how?) in the province, and searched for that at the pharmacies? If so, that isn’t that interesting if there are other fairly low-priced generics which are available instead. and it is understandable that that particular brand was not always available. Or in each pharmacy, did they identify the lowest priced generic carried by that particular pharmacy, which wasn’t always available? What is there was another fairly low priced generic available instead? There is a big different in policy implications between no generic alternative being available at all, or another one being available at a slightly higher price.

Q: One issue for me is that “Lowest Priced Generic” was not clearly defined. Does this mean that the authors identified a lowest priced generic brand overall (how?) in the province, and searched for that at the pharmacies? If so, that isn’t that interesting if there are other fairly low-priced generics which are available instead. and it is understandable that that particular brand was not always available.

A: We studied the price of LPG in public medical institutions and pharmacies in Zhejiang Province through questionnaire surveys, processed the data strictly in accordance with the WHO/HAI method, and presented the obtained data on the manuscript. We were unable to investigate every public hospital and pharmacy in Zhejiang Province, so we adopted a sampling survey method. Our research results are sample data.

Q: Or in each pharmacy, did they identify the lowest priced generic carried by that particular pharmacy, which wasn’t always available? What is there was another fairly low priced generic available instead? There is a big different in policy implications between no generic alternative being available at all, or another one being available at a slightly higher price.

A: In the pharmacies we investigated, if there is no OB, a generic drug of the same name and dosage form will be used as LPG for research. If there is no generic drug with the same name and dosage form, there is no universal substitute at all, and it will be displayed as "0" in the table, which will result in poorer availability results.

Revision:During the research process, we surveyed and collected the prices and availability of OB and LPG for each of the 50 drugs. OB is the originator brand, and LPG is the lowest-priced generic. Originator brand (OB) and lowest-priced generic equivalent (LPG) medicines were monitored and these prices were then compared with international reference prices (IRPs). This study adopted the standard sampling survey method provided by WHO/HAI to determine the lowest price of "LPG". Our research focuses on sample surveys, and surveys are conducted in the form of questionnaires. In the hospitals and pharmacies surveyed, if there is no OB, a generic drug with the same name and dosage form will be used as LPG for research. If there is no generic drug with the same name and dosage form, there is no universal substitute, and it will be displayed as "0" in the table.

3. Abstract: I was puzzled as to why Methods, Findings and Conclusions were repeated twice. Perhaps one was original, one was revised? In any case, under Findings, line 4 (both versions): suggest refer to “innovator brands” instead as “OBs” – confusing to the reader to call the same thing by two different names.

Modifications have been made in the revised version

4. Introduction, para 2: don’t mention the “three in one” policy without a brief explanation – readers will not all be familiar with this. Readers will also not understand what the significance of the “province’s unification” is without explanation.

We have deleted “three in one” policy

5. In many places a space is missing: I will list the words and suggest the authors do a “search and replace”: “anadditional” “mainurban” “theprice” “andcollected” forms.No” “Taizhou,Jiaxing” “Zhejian:one” “ratios(MPRs)” “brands(OBs)” “generics(LPGs)” “work .For” “expenses, [15]and” “studywas”

Thank you very much for the reviewer’s reading. We have corrected the error and marked it in red

6. Table 2: this is a continuation of Table 1, and should have the title “Table 1 (continued)” or similar, and subsequent tables should accordingly be renumbered.

We have renumbered the table

7. Table 3: suggest add “n=50” to the title for clarity

We have add “n=50”

8. Table 7: in the column titles, clarify in a footnote perhaps that “Duration” means “duration of treatment course over a one-month period” or similar.

We made a mark under the revised "Table 6"

9. Discussion: second full paragraph discusses “the government’s inadequate subsidies for essential medicines..” – is what is meant that the government does not permit any handling fee for medicines by pharmacies? (That is indeed a problem regarding incentives).

In China, pharmacies do not charge prescription review and preparation service fees for patients selling drugs, which is very different from the pharmacy operation model in the United States and other countries. The Chinese government has substantial financial subsidies for public hospitals, but not for pharmacies. The current latest policy is that when the government requires retail pharmacies included in medical insurance to sell drugs in the government's centralized procurement catalog, the price can be 15% higher than the price of public hospitals, and the higher part can be paid by medical insurance, thereby indirectly subsidizing retail pharmacies.

Revision: Secondly, The government's essential drugs policy prohibits the sale of essential drugs by medical institutions at premium prices, and through allocating funds to hospitals to maintain their operations, but government subsidies usually only account for 10% of operating expenses. It can be said that hospital pharmacies are now the pure cost expenditure units of medical institutions, so they tend to reduce the scale of drug supply. In China, social pharmacy do not charge prescription review and preparation service fees for drugs sold to patients，its profit comes from the premium between the purchase and sale of medicines, which is very different from how pharmacies in the United States and other countries operate.

10. Same paragraph: “.Although hospitals have charged for pharmaceutical services..” (insert underlined word). Also “…pharmaceutical services in Zhejiang or China are in their infancy..” (not “is”)

The essential drug list is an important guideline for the implementation of the essential drug system. China has implemented the essential drug system for several years. Primary medical institutions have basically achieved full coverage of essential drugs. With the advancement of the medical and health system reform, the next goal of the essential drug system is inevitable. It is to increase the use rate of essential medicines in secondary and tertiary hospitals.

11. Paragraph beginning: “The essence of "4 + 7" is to exchange price by quantity.” I don’t understand what the authors mean here. Is what they are saying “The essence of “4+7” is to use larger volumes of purchase to drive down prices”.

The so-called "4+7" policy is the abbreviation of China's pharmaceutical industry, and its true meaning is the "National Organized Drug Centralized Procurement and Use Pilot Program". The Chinese government officially implemented the centralized drug procurement policy on January 17, 2019. The original intention of this policy is to use bulk purchases to keep prices down.

Revision:On January 17, 2019, the Chinese government issued the "National Organized Drug Centralized Procurement and Use Pilot Program", marking the beginning of the Chinese government's formal implementation of the centralized drug procurement policy, which has greatly reduced the prices of drugs in China. The abbreviation of this policy is called "4+7" centralized procurement policy.The Chinese government forces the bid-winning companies to lower the price of medicines. The bid-winning medicines of the bid-winning companies can gain more market share and lower the price of medicines through large purchases.

12. “but also has its own policies among various provinces” – not clear what is meant here. Are the authors trying to say “but each province has its own policies”. “The policies of Zhejiang Province encourage public and private pharmacies to increase the availability of medicines and
reduce the affordability of medicines.”. How?

In China, each province has its own policies, and the provincial government will respond to the official documents of the Chinese government and issue relevant policies based on the development of its own province.

Revision: In China, each province has its own policies, and the provincial government will respond to the official documents of the Chinese government and issue relevant policies based on the development of its own province. For example, on 12th and 6th, 2019, the National Medical Security Administration of China issued the "Opinions on Doing a Good Job in Current Drug Price Management". Article 1 of Chapter 3 mentioned that for shortage of drugs, operators are allowed to quote their prices and directly online. The medical institution purchases at the online price or further negotiates with the operator for purchase. On June 28, 2020, the Zhejiang Provincial Government issued the “Zhejiang Province to Enhance the Function of the Centralized Pharmaceutical Procurement Platform to Promote the Reform of the Full Coverage of Medical Insurance and Drug Payment Standards,” Chapter III mentioned that for clinical needs, public medical institutions are allowed to negotiate with enterprises in accordance with the principle of fairness to determine the purchase price within the limit of 1% of the settlement amount of drugs in the previous year, and purchase products by themselves through the provincial drug equipment purchase platform, if there is no online traded products within the platform or they cannot be purchased at the set price within the platform. This reflects the exploration of the self-procurement system of public medical institutions.

13. Our research is based on the actual situation of Zhejiang Province, China, and is different from other countries. Other countries do not have the same policies as China, so the research results are also different.” This may be true but is too vague to be useful.

Your opinion is very good. The original text has been deleted

14. Next page: “Due to confidentiality reasons, some data have errors and missing” – what confidentiality reasons? Please explain

In fact, we have obtained the right to publish research data publicly, and we have organized the research data into a compressed package and uploaded it to the submission system. Inside is our questionnaire, because it is raw data, it is all in Chinese.

15. Conclusion: “the government should increase investment” – in what? People don’t usually consider providing a pharmacy service fee for medicines, as investment.

Our original intention was to say that the government should increase subsidies in the field of public health care, mainly subsidizing retail pharmacies, as mentioned above.

Revision: First, the government should increase subsidies for health insurance. In terms of the development trend, investment in this field has been increasing in recent years. For example, in 2019, the income of the basic medical insurance fund for Chinese workers and urban and rural residents increased by 9.94% year on year, and the income of the basic medical insurance fund for urban and rural residents increased by 7.71% year on year. Secondly, the Government should further play the role of social pharmacies in improving the availability and affordability of essential medicines. Now, some of the measures, like lowering the barriers for pharmacies to enter the health care system, and allowing them to sell essential drugs at higher prices than medical institutions, we think it should be effective. Third, allowing pharmacists in social pharmacies to charge pharmaceutical service fees will significantly improve the income structure of social pharmacies and encourage pharmacists to tend to provide more types of drugs and better pharmaceutical care, instead of only paying attention to drugs with high premium prices. The good news is that pharmacists' drug preparation service fee may be recognized by law. June 18, 2020, the Chinese government issued the pharmacist law of the People's Republic of China second draft (draft) ", stipulated in article 5 of pharmacists to provide pharmaceutical professional technical services to patients with diagnosis and treatment of behavior belong to shall, through the proper way to manifest the value of their labor, which indicated that pharmacists service charge is likely to become a reality in the future.

16. Thank you for the effort towards improving the ms. However, it is very difficult to know if changes were clearly made or points were inserted in the revised version of the ms. Changes were neither highlighted nor indicated by track changes.

Conclusions - Authors are supposed to conclude based on the objectives and main findings. But, the last paragraph, the authors have included a ref i.e. Minghuan Jiang et al, and it looks like a discussion section.
Figure 1 - cetirizine - small letter 'c'. The authors mentioned that this typo had been corrected.

I doubt that proper actions have been taken and please highlighted the corrections and improvements in the revised ms.

We have deleted this sentence” Affordability found in the present study was better than the study conducted in Shaanxi by Minghuan Jiang et al, which also showed that most medicines for these common diseases were affordable.”because we feel it inappropriate.

The chart has been modified
