# Supplementary material for: Availability, prices and affordability of essential medicines in Zhejiang Province, China
Source: PLoS One. 2020 Nov 24;15(11):e0241761. doi: 10.1371/journal.pone.0241761 (PMC7685453; doi:10.1371/journal.pone.0241761)
Supplement: Attachment — Submitted filename: Response to Reviewers(2020.9).docx [file pone.0241761.s004.docx]

**Rebuttal letter**

Dear Susan Horton
Thanks you very much for your comments and suggestions.

As suggested, We have made amendments to the academic papers, in accordance with the requirements of the academic editors, and responded to the comments made by the reviewers. The details are attached below. We have asked editge to help us polish the language and correct the previous language problem.

We have revised the manuscript, according to the comments and suggestions of reviewers and editor, and responded, point by point to, the comments as listed below. Some of the references we cited are published in domestic academic journals in China and may not be found in foreign databases.

I would like to re-submit this revised manuscript to PLOS ONE, and hope it is acceptable for publication in the journal.

Looking forward to hearing from you soon.

With kindest regards.

Yours Sincerely

Guojun Sun

Replies to Reviewers and Editor

First of all, we thank both reviewers and editor for their positive and constructive comments and suggestions.

Replies to Reviewer #1:

1.Thank you for responding to the previous comments. There is one outstanding issue. "Lowest priced generic" is still not well-defined. Is this one particular brand which is deemed the lowest-priced in the province? (this is what it seems to be)? If not, please define out of which subset it is "lowest priced".

During the study, we investigated and collected the prices of Originator Brands (OBs) and Lowest-priced Generics (LPGs) for 50 drugs and then compared these prices with international reference prices (IRPs). OBs are products of the same specification produced by the originator manufacturer of the drug, and LPGs are the cheapest generic drugs found in the research institute. This study adopted the standard sampling survey method provided by the WHO/HAI to determine the cheapest LPGs. Our research focuses on sample surveys, and surveys are conducted in the form of questionnaires. In the hospitals and pharmacies surveyed, if there is no OB, a generic drug with the same name and dosage form will be used as an LPG for research. If there is no generic drug with the same name and dosage form, there is no universal substitute, and it will be displayed as “0” in the table.

2.Thank you for explaining the policy in the conclusions more fully. Unfortunately the language of the draft is unacceptable at the moment. At this point you need to pay someone for whom English is their first language and who has some understanding of the subject matter to edit the draft. This will be the third revision of this article. I strongly recommend that you perfect the language this time since I do not know if the journal will accept further revisions (this is up to the editor).

We have asked editge to help us polish the language and correct the previous language problem.
