# Supplementary material for: Availability, prices and affordability of essential medicines in Zhejiang Province, China
Source: PLoS One. 2020 Nov 24;15(11):e0241761. doi: 10.1371/journal.pone.0241761 (PMC7685453; doi:10.1371/journal.pone.0241761)
Supplement: Attachment — Submitted filename: Response to Reviewers(2020.10).docx [file pone.0241761.s005.docx]

**Rebuttal letter**

Dear Susan Horton
Thanks you very much for your comments and suggestions.

As suggested, We have made amendments to the academic papers, in accordance with the requirements of the academic editors, and responded to the comments made by the reviewers. We have made changes based on the previous manuscript.The details are attached below. We have asked editge to help us polish the language and correct the previous language problem.

We have revised the manuscript, according to the comments and suggestions of reviewers and editor, and responded, point by point to, the comments as listed below.

I would like to re-submit this revised manuscript to PLOS ONE, and hope it is acceptable for publication in the journal.

Looking forward to hearing from you soon.

With kindest regards.

Yours Sincerely

Guojun Sun

Replies to Reviewers and Editor

First of all, we thank both reviewers and editor for their positive and constructive comments and suggestions.

Replies to Reviewer #1:

Thank you for getting help with the English. Now that I can understand better what you are trying to say, I can now offer the following edits so that the readers of the journal will also understand. I recommend the following edits. Since there do not seem to be page or line numbers, I have numbered according to the page number in the proof which overall has 53 pages.

Thank you for your patience in reviewing the manuscript, we have revised the manuscript again.

P13 of 53: “LPGs are the cheapest generic drugs found in the research institute” Do you mean “LPGs are the cheapest generic drugs found by the University research institute”?

In our research, LPGs are the cheapest generic drugs found by the University research institute.

p13 pf 53 “Data collection included six cities in Zhejiang Province. Hangzhou was identified as the major urban center of Zhejiang, and an additional five cities were chosen randomly. As a result, six cities were selected: Hangzhou, Ningbo, Shaoxing, Huzhou, Taizhou, and Jiaxing”. Delete this sentence – duplicates statement previously. In following sentence say “area surveyed” not “surveyed area”

already edited

In several places the authors say “We refer to Table X”. This is unnecessary, as long as the table is cited in the text.

already edited

P17 of 53, last 2 lines (last 2 lines of Table 2). These should be deleted.
P19 of 53. Restate the following sentence: ,”The results in Table 5 show that the patient price in the public sector is 13.41 lower than that in the private sector for OBs, while it is 5.21 higher for LPGs.” As “,The results in Table 5 show that the patient price in the public sector is 13.41 for OBs, lower than that in the private sector while for LPGs it is 5.21, higher than that in the private sector “.

already edited

P20 of 53: “treating peptic ulcer with omeprazole required 5.2 days’ wages.” Treating for how long (the recommended course is 4-8 weeks).
already edited

P21, last line. “the mean availability of OBs and LPGs was 12.9% and 29.5%, respectively.[11].” Is this in private pharmacies? If so, state that.
already edited

P22 of 53, last line. Does “social” pharmacies mean “public”? If so, make this change from here onwards (used several times).
already edited

P23 of 53: I think instead of “4+7 belt quantity purchasing policy” what is means is “4+7” bulk purchasing policy. State that.
already edited

P23 of 53, last line. If the average price of medicines was reduced by more than 60%, what exactly was reduced by 92%?
already edited

P24 of 53, line 4: instead of “burden of patients” say “burden on patients”.
already edited

P24. Delete entire paragraph (duplicates material elsewhere “The essence of “4+7” policy is to use larger purchase volumes to drive down prices. Successful bidders who pass the conformity evaluation of generic drugs willgreatly reduce the price of drugs. The government will give the winning bidder a certain market share. The coverage of high-quality and low-price generic drugs will expand; the pressure to make drugs affordable will be reduced; and patients will have
better drug choice.[19Some pharmaceutical industry associations apply for
implementation of the policy to be suspended, but the decision of the health insurance
department to implement the policy is very resolute. Because of their own interests,
pharmaceutical enterprises will strictly control the quantity of drugs produced,
resulting in a rapid reduction in the availability of drugs.[20]”

already edited

p24. Also delete sentence “In China, each province has its own policies, and
the provincial government will respond to the official documents of the Chinese
government and issue relevant policies based on the development of its own province.” Since this simply restates what was said in the previous sentence.
already edited

P25: delete “This reflects the examination of the self-procurement system of public
medical institutions. In this study, we adopted a systematic research method to study related issues, combined with the actual situation and policies to study the availability andaffordability of drugs in public and private pharmacies in Zhejiang”. This simply restates what has been previously said.
already edited

P26: delete “The availability and affordability of medicines is a complex systemic problem.The question first needs to clarify the essential causes of the shortage of drugs; on thisbasis, grasp the key factors; and propose solutions in regard to drug prices, marketenvironment, policy systems, and legislation. The smooth implementation of thesesolutions also requires the coordination and cooperation of various governmentdepartments and stakeholders of the drug supply. Only when the main bodies of thedrug supply chain work together can the current issues of great concern be solved tothe greatest extent.” Also repetitious.

already edited

P26: Delete “In terms of the development trend,” (repetitious)
already edited

P26: what is meant by “the income of the basic medical insurance fund”? Does this mean aggregate income of the fund in the province, or does it mean “each worker’s basic medical insurance allotment”
already edited

P26: “Second, the government should further play the role of social
pharmacies in improving the availability and affordability of essential medicines”. Suggest restate as “Second, the government should improve the availability and affordability of essential medicines in public pharmacies”.
already edited

P27. Delete “with diagnosis and treatment of behavior belonging to shall, through the
proper way to manifest the value of their labor”: sentence is clearer without this.
already edited
